# Ahmpatinin ^i^Bu, a new HIV-1 protease inhibitor, from *Streptomyces* sp. CPCC 202950†

**DOI:** 10.1039/c7ra13241g

**Published:** 2018-01-30

**Authors:** Ming-Hua Chen, Shan-Shan Chang, Biao Dong, Li-Yan Yu, Ye-Xiang Wu, Ren-Zhong Wang, Wei Jiang, Zeng-Ping Gao, Shu-Yi Si

**Affiliations:** Institute of Medicinal Biotechnology, Chinese Academy of Medical Sciences, Peking Union Medical College Beijing 100050 China sisyimb@hotmail.com; School of Chinese Materia Medica, Beijing University of Chinese Medicine Beijing 100102 China gaozp@bucm.edu.cn; Key Laboratory for Uighur Medicine, Institute of Materia Medica of Xinjiang Urumqi 830004 China

## Abstract

Ahmpatinin ^i^Bu (1) and statinin ^i^Bu (2), two new linear peptides, a novel pyrrolidine derivative, (−)-(*S*)-2-[3-(6-methylheptanamido)-2-oxopyrrolidin-1-yl] acetic acid (3), and three known pepstatin derivatives (4–6) along with their corresponding methanolysis artifacts (7–9) were isolated from *Streptomyces* sp. CPCC 202950. Their structures were elucidated on the basis of extensive spectroscopic data using Marfey's analysis, chiral-phase HPLC, and ECD and OR calculation to determine the absolute configurations. Compound 1 contains an unusual amino acid, 4-amino-3-hydroxy-5-(4-methoxyphenyl)pentanoic acid (Ahmppa), and 3 is the first natural product with a 2-(3-amino-2-oxopyrrolidin-1-yl)acetic acid system. Compounds 1, 2, and 4–9 are HIV-1 protease inhibitors. In particular, ahmpatinin ^i^Bu (1) exhibits significant inhibitory activity against HIV-1 protease with an IC_50_ value of 1.79 nM. A preliminary structure–activity relationship is discussed.

## Abstract 2

Ahmpatinin ^i^Bu and statinin ^i^Bu, two new linear peptides, were isolated from *Streptomyces* sp. CPCC 202950. Ahmpatinin ^i^Bu exhibited significant inhibitory activity against HIV-1 protease with an IC_50_ value of 1.79 nM.

## Graphical abstract

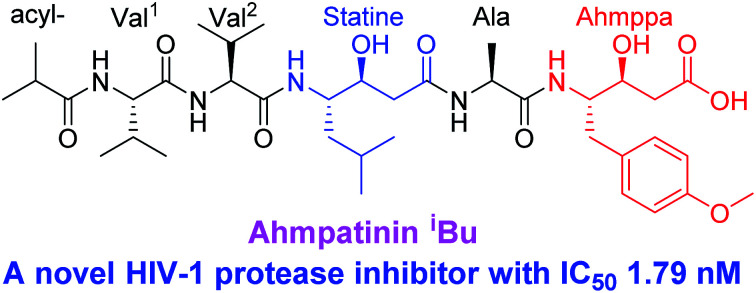

## Introduction

The most effective treatment for HIV infection, HAART (Highly Active AntiRetroviral Therapy) that including several HIV-1 protease inhibitors combination with reverse transcriptase inhibitors, and other inhibitors with integrase, membrane fusion, and viral attachment, have significantly reduced the mortality of AIDS patients and improved the quality of life of those infected with HIV.^1–4^ However, according to World Health Organization and Joint United Nations Program on HIV/AIDS data, in 2016, there were still 36.7 million people living with HIV, 1.8 million newly infected and 1.0 million HIV-related deaths.^5,6^ Therefore, AIDS is still a substantial threat to global public health. HIV-1 protease (HIV-1 PR) is an essential enzyme in the life cycle of HIV and has been used as a promising target for AIDS therapy.^7–9^ Although 11 HIV-1 PR inhibitors such as amprenavir, indinavir, nelfinavir, ritonavir, saquinavir, and tipranavir have been approved by the FDA and applied extensively in clinical evaluations,^10,11^ drug toxicity, resistance, and drug–drug interactions remain serious problems for HIV/AIDS treatments,^1,12–14^ prompting us to discover new anti-HIV drugs.

**Fig. 1:**
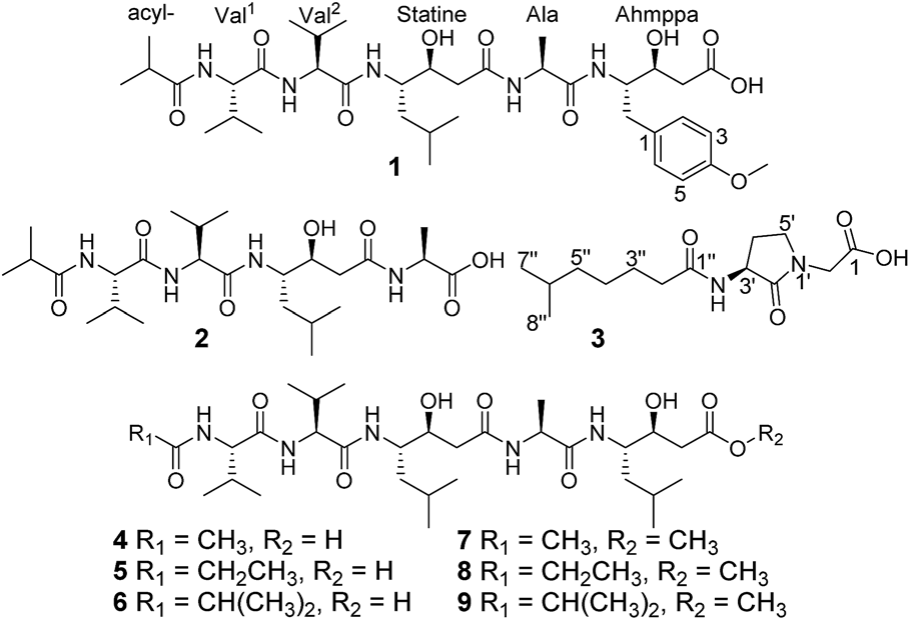
Chemical structures of compounds 1–9.

In our previous report, a new HIV-1 protease inhibitor named 4862F^15^ and 11 known compounds^16^ were isolated from the liquid fermentation of the strain *Streptomyces* sp. CPCC 202950. Unexpectedly, the crude extract of the rice culture of *Streptomyces* sp. CPCC 202950 also significantly inhibited HIV-1 protease activity with an IC_50_ value of 20.0 ng ml^−1^. Extensive investigation of the secondary metabolites of the rice culture extract of the strain resulted in the isolation of two new linear peptides, ahmpatinin ^i^Bu (1) and statinin ^i^Bu (2), a new pyrrolidine derivative, (−)-(*S*)-2-[3-(6-methylheptanamido)-2-oxopyrrolidin-1-yl]acetic acid (3), and three known pepstatin derivatives along with their corresponding methanolysis artifacts (4–9) (Fig. 1). Among them, compound 1 contains an unusual amino acid, 4-amino-3-hydroxy-5-(4-methoxyphenyl)pentanoic acid. Compound 3 represents the first natural product with a 2-(3-amino-2-oxopyrrolidin-1-yl)acetic acid system, which was synthesized in a previous study.^17^ Herein, we report the isolation, structural elucidation, and biological activities of these compounds.

## Results and discussion

Ahmpatinin ^i^Bu (1) was isolated as a white powder and was determined to be C_37_H_61_N_5_O_10_ on the basis of positive HRESIMS, indicating 10 degrees of unsaturation. The ^1^H NMR spectrum of 1 (Table 1) in DMSO-*d*_6_ exhibited characteristics of a typical peptide, with five NH protons (*δ*_H_ 7.4–7.9) and five amino acid α-protons (*δ*_H_ 3.8–4.3). Furthermore, *para*-disubstituted aromatic ring protons [*δ*_H_ 6.80 (2H, d, H-3 and H-5) and 7.12 (2H, d, H-2 and H-6)], two oxygenated methine protons (*δ*_H_ 3.82 and 3.85), four methylene protons, four methine protons, and a methoxyl singlet [*δ*_H_ (3.70, s, 3H)] along with nine additional methyl doublets (*δ*_H_ 0.7–1.2) were also observed in the ^1^H NMR spectrum. Six carbonyl signals (*δ*_C_ 170–176) and five α-amino acid carbon signals (*δ*_C_ 48–58) were observed in the ^13^C NMR spectrum, further indicating that 1 was a peptide. Analysis of the DEPT and ^13^C NMR spectra revealed additional 1,4-disubstitued benzene ring groups (*δ*_C_ 113–158), ten methyl groups, four methylenes, and six sp^3^ methines.

**Table 1:** NMR spectroscopic data of 1 and 2^a^

	No.	1		2	
		*δ*_H_	*δ*_C_, type	*δ*_H_	*δ*_C_, type
Val^1^	CO		171.1, C		171.0, C
	α	4.14, dd (7.8, 8.4)	57.7, CH	4.14, dd^b^	57.7, CH
	β	1.96, m	30.1, CH	1.94, m	30.1, CH
	γ	0.81, d(6.6)	18.4, CH_3_	0.81, d(6.6)	18.2, CH_3_
	γ′	0.81, d(6.6)	19.8, CH_3_	0.81, d(6.6)	19.8, CH_3_
	NH	7.79, d(8.4)		7.78, d(9)	
Val^2^	CO		170.6, C		170.6, C
	α	4.14, dd(7.8, 8.4)	57.8, CH	4.14, dd^b^	57.8, CH
	β	1.96, m	30.4, CH	1.94, m	30.5, CH
	γ	0.81, d(6.6)	18.2, CH_3_	0.81, d(6.6)	18.1, CH_3_
	γ′	0.83, d(6.6)	19.4, CH_3_	0.83, d(6.6)	19.4, CH_3_
	NH	7.71, d(9)		7.68, d(9)	
Sta	CO		170.7, C		170.8, C
	α	2.11, m	39.2, CH_2_	2.10, m	39.5, CH_2_
	β	3.82, m	69.0, CH	3.81, m	69.0, CH
	γ	3.82, m	50.7, CH	3.85, m	50.7, CH
	δ	1.34, m	38.7, CH_2_	1.32, m	38.5, CH_2_
		1.25, m		1.25, m	
	ε	1.52, m	24.2, CH	1.52, m	24.2, CH
	ζ	0.78, d(6.6)	21.6, CH_3_	0.78, d(6.6)	21.6, CH_3_
	ζ′	0.83, d(6.6)	23.4, CH_3_	0.83, d(6.6)	23.5, CH_3_
	NH	7.47, d(9)		7.50, d(9)	
Ala	CO		172.1, C		174.2, C
	α	4.22, m	48.2, CH	4.17, dq^b^	47.4, CH
	β	1.12, d(7.2)	18.0, CH_3_	1.23, d(7.8)	17.2, CH_3_
	NH	7.85, d(7.2)		8.07, d(7.2)	
Ahmppa	CO		172.8, C		
	α	2.23, dd(15.6, 3.6)	38.8, CH_2_		
		2.16, dd(15.6, 8.4)			
	β	3.85, m	67.2, CH		
	γ	3.83, m	54.4, CH		
	δ	2.75, dd(13.8, 6)	35.4, CH_2_		
		2.56, dd(13.8, 8.4)			
	1		130.9, C		
	2	7.12, d(8.4)	130.0, CH		
	3	6.80, d(8.4)	113.5, CH		
	4		157.5, C		
	5	6.80, d(8.4)	113.5, CH		
	6	7.12, d(8.4)	130.0, CH		
	NH	7.53, d(9)			
	OMe-4	3.70, s	54.9, CH_3_		
Acyl	CO		176.2, C		176.2, C
	α	2.51, m	33.6, CH	2.52, m	33.6, CH
	β	0.95, d(6.6)	19.2, CH_3_	0.95, d(6.6)	19.2, CH_3_
	β′	0.98, d(6.6)	19.3, CH_3_	0.98, d(6.6)	19.3, CH_3_

a NMR data (*δ*) were measured at 600 MHz for ^1^H and at 150 MHz for ^13^C in DMSO-*d*_6_. Proton coupling constants (*J*) in Hz are given in parentheses. The assignments were based on ^1^H–^1^H COSY, HSQC, and HMBC experiments.

b The *J*-value was not determined due to overlapping signals.

**Fig. 2:**
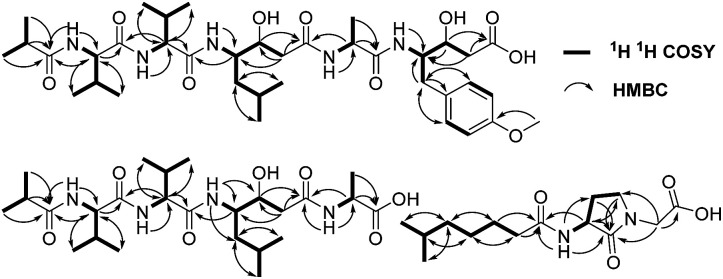
The key ^1^H–^1^H COSY and HMBC correlations of 1–3.

**Fig. 3:**
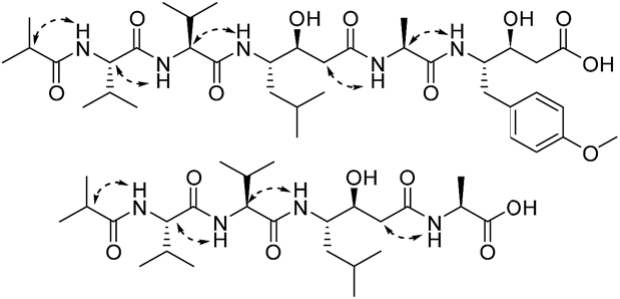
The key NOESY correlations of 1 and 2.

A comparison of the NMR spectra of compounds 1 and ahpatinin ^i^Bu^18^ revealed that the mono-substituted benzene moiety in ahpatinin ^i^Bu was replaced with a para-disubstituted aromatic ring group in 1. This was confirmed by 2D NMR (Fig. S6–S9†). HMBC correlations (Fig. 2) of H-4-OMe and C-4, δ-H-Ahmppa/C-1, C-2, C-6, C-β-Ahmppa and C-γ-Ahmppa, α-H-Ahmppa/C-β-Ahmppa, C-γ-Ahmppa and C-CO-Ahmppa, β-H-Ahmppa and C-CO-Ahmppa, and NH-Ahmppa and C-γ-Ahmppa indicated the presence of a 4-amino-3-hydroxy-5-(4-methoxyphenyl)pentanoic acid (Ahmppa) residue. Integrated 2D NMR (^1^H NMR, COSY, HSQC, and HMBC) data were used to assign five partial structures consisting of the amino acids alanine, two valines, statine (Sta), and an isobutyl (^i^Bu) group. HMBC correlations from α-H-Val^1^ (*δ*_H_ 4.14) and NH-Val^1^ (*δ*_H_ 7.79) to ^i^Bu-C=O (*δ*_C_ 176.2), α-H-Val^2^ (*δ*_H_ 4.14) to Val^1^-C=O (*δ*_C_ 171.1), γ-H-Sta (*δ*_H_ 3.82) to Val^2^-C=O (*δ*_C_ 170.6), α-H-Ala (*δ*_H_ 4.22) to Sta-C=O (*δ*_C_ 170.7), and γ-H-Ahmppa (*δ*_H_ 3.83) to Ala-C=O (*δ*_C_ 172.1) established the sequence ^i^Bu–Val^1^–Val^2^–Sta–Ala–Ahmppa, which was verified by NOESY correlations (Fig. 3 and S9†) from NH-Val^1^ (*δ*_H_ 7.79) to α-H-^i^Bu (*δ*_H_ 2.51), NH-Val^2^ (*δ*_H_ 7.71) to α-H-Val^1^ (*δ*_H_ 4.14), NH-Sta (*δ*_H_ 7.47) to α-H-Val^2^ (*δ*_H_ 4.14), NH-Ala (*δ*_H_ 7.85) to α-H-Sta (*δ*_H_ 2.11), and NH-Ahmppa (*δ*_H_ 7.53) and α-H-Ala (*δ*_H_ 4.22).

The absolute configurations of Ala and Val in 1 were determined to be L using Marfey's method (Fig. S27† and Experimental section),^19,20^ whereas the Ahmppa residue was established to be 3*S*, 4*S* by chiral-phase HPLC after acid hydrolysis in comparison to synthetic standards (Fig. S28† and Experimental section). In addition, the acid hydrolysis of 1 followed by HPLC purification gave statine, and its ^1^H NMR data agreed with the synthetic standard of (3*S*,4*S*)-statine. Meanwhile, the specific rotation {[*α*]^20^_D_ = −19.5 (*c* 0.05, H_2_O)} of the statine from the hydrolysis of 1 was consistent with that of (3*S*,4*S*)-statine {[*α*]^20^_D_ = −20.4 (*c* 0.502, H_2_O)}.^21^ Therefore, the final structure of 1 was determined to be ^i^Bu-l-Val^1^-l-Val^2^-(3*S*,4*S*)-Sta-l-Ala-(3*S*,4*S*)-Ahmppa and was named ahmpatinin ^i^Bu.

Compound 2 was obtained as a white amorphous powder, and the molecular formula was assigned as C_25_H_46_N_4_O_7_ by HRESIMS based on a [M − H]^−^ ion at 513.3275 (calc. 513.3283). The MS data for 2 indicated the absence of the Ahmppa moiety relative to compound 1. Extensive analysis of 2D NMR (^1^H–^1^H COSY, HSQC, HMBC, and NOESY) spectroscopic data (Fig. S15–S18†) revealed the sequence of ^i^Bu–Val^1^–Val^2^–Sta–Ala. The absolute configurations of the amino acid residues and the statine unit were also confirmed to be l-Ala, l-Val and (3*S*,4*S*)-statine using the same protocol as 1. Hence, compound 2 was deduced to be ^i^Bu-l-Val^1^-l-Val^2^-(3*S*,4*S*)-Sta-l-Ala and was named statinin ^i^Bu.

**Fig. 4:**
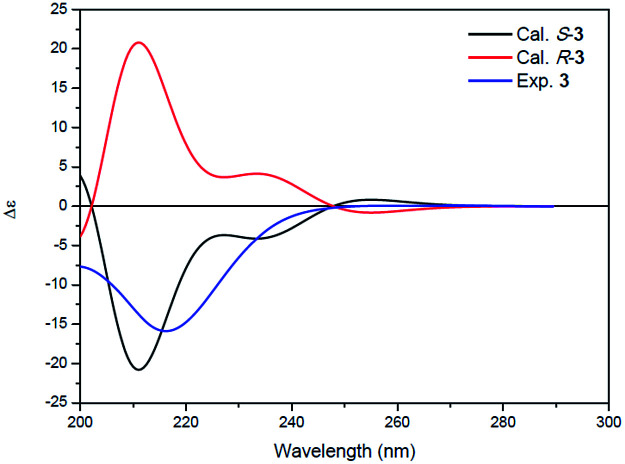
Experimental ECD spectrum of 3 (blue) and the calculated ECD spectra of (*R*)-3 (red) and (*S*)-3 (black).

Compound 3 was isolated as a white powder. Its molecular formula C_14_H_24_N_2_O_4_, requiring four degrees of unsaturation, was established from the HRESIMS data (*m*/*z* 285.1802 [M + H]^+^, calcd for C_14_H_25_N_2_O_4_, 285.1808). The IR spectrum of 3 displayed the presence of hydroxy and/or amino (3287 cm^−1^), carboxyl (1700 cm^−1^) and amide (1645 cm^−1^) functionalities. Analysis of the ^1^H NMR, ^13^C NMR and HSQC spectra (Table 2) of 3 revealed two doublet methyls, seven sp^3^ methylenes, two sp^3^ methines, and three carbonyls (*δ*_C_ 170.0, 172.3, and 172.4). The ^1^H–^1^H COSY cross-peaks of H_2_-2′′/H_2_-3′′/H_2_-4′′/H_2_-5′′/H-6′′/H_3_-7′′(H_3_-8′′) and HMBC correlations of H_3_-7′′(H_3_-8′′)/C-6′′ and C-5′′; H-6′′/C-4′′, C-5′′, C-7′′ and C-8′′; H_2_-5′′/C-3′′, C-4′′, C-6′′, C-7′′ and C-8′′; H_2_-4′′/C-2′′, C-3′′, C-5′′ and C-6′′; H_2_-3′′/C-1′′, C-2′′, C-4′′ and C-5′′; and H_2_-2′′/C-1′′, C-3′′ and C-4′′ indicated the presence of a 6-methylheptanoyl moiety in 3. Meanwhile, in conjunction with the degrees of unsaturation and the molecular composition, ^1^H–^1^H COSY correlations of NH/H-3′/H-4′/H-5′ along with HMBC correlations of NH/C-2′, C-3′ and C-4′; H-3′/C-2′ and C-4′; H_2_-4′/C-2′, C-3′ and C-5′; H_2_-5′/C-2′, C-3′ and C-4′; and H_2_-2/C-1, C-2′ and C-5′ demonstrated the presence of a 2-(3-amino-2-oxopyrrolidin-1-yl)acetic acid moiety.^17^ Finally, the HMBC correlation from H-3′ and NH to C-1′′ suggested that the two moieties were connected *via* an amide bond. Thus, compound 3 was confirmed to be 2-(3-(6-methylheptanamido)-2-oxopyrrolidin-1-yl)acetic acid. The absolute configuration at C-3′ was determined by comparing the experimental circular dichroism (CD) spectrum and optical rotation (OR) data with the computed ECD and OR data. A comparison of the experimental CD spectrum with the calculated ECD spectrum of 3 (Fig. 4) demonstrated that compound 3 possessed an *S* configuration, which was verified by the computed OR, which was −41.5 for (*S*)-3. The experimental OR value was −100.5 in acetonitrile. Therefore, compound 3 was elucidated to be (−)-(*S*)-2-[3-(6-methylheptanamido)-2-oxopyrrolidin-1-yl]acetic acid.

**Table 2:** NMR spectroscopic data of 3^a^

No.	3	
	*δ*_H_	*δ*_C_, type
1		170.0, C
2	3.99, d(17.4)	44.1, CH_2_
	3.89, d(17.4)	
2′		172.4, C
3′	4.41, m	49.5, CH
4′	2.29, m	26.2, CH_2_
	1.76, m	
5′	3.33, m	43.8, CH_2_
1′′		172.3, C
2′′	2.10, t(7.2)	35.3, CH_2_
3′′	1.47, m	25.5, CH_2_
4′′	1.25, m	26.4, CH_2_
5′	1.14, m	38.2, CH_2_
6′′	1.51, m	27.3, CH
7′′	0.84, d(6.6)	22.5, CH_3_
8′′	0.84, d(6.6)	22.5, CH_3_
NH	8.13, d(8.4)	

a NMR data (*δ*) were measured at 600 MHz for ^1^H and at 150 MHz for ^13^C in DMSO-*d*_6_. Proton coupling constants (*J*) in Hz are given in parentheses. The assignments were based on ^1^H–^1^H COSY, HSQC, and HMBC experiments.

In addition to compounds 1–3, the known pepstatin Ac (4),^18,22^ pepstatin Pr (5),^18,22^ pepsinostreptin (6),^18^ pepstatin Ac methyl ester (7), pepstatin Pr methyl ester (8), and pepsinostreptin methyl ester (9) were also isolated and identified from the strain *Streptomyces* sp. CPCC 202950. Compounds 4–6 incubated with methanol showed the presence of corresponding methyl esters 7–9, respectively. These results indicated that 7–9 were indeed artifacts derived from methanolysis during the isolation process. Furthermore, a new benzamide analog and seven known compounds were isolated from the inactive fractions of the crude extract in our previous report.^23^

Pepstatin and ahpatinin derivatives have been shown to exhibit significant inhibitory activity against aspartic protease.^18,24–28^ HIV-1 PR is a 22 kDa dimeric aspartyl protease. As expected, compounds 1 and 2 along with pepstatin derivatives 4–9 from the active fractions showed significant inhibitory activity against HIV-1 protease with IC_50_ values of 1.79 nM to 3.19 μM, while compound 3 was inactive (the positive control indinavir gave an IC_50_ value of 1.82 nM) in preliminary *in vitro* assays (Table 3). A comparison of the activities of 4–6 and 7–9 demonstrated that the exposed terminal carboxyl group was very important for the HIV-1 protease inhibitory activity. Analysis of the inhibitory abilities of 1 and 4–9 indicated that the Ahmppa unit was better than the statine moiety. Compound 2 showed weaker inhibitory ability than 1 and 4–9, suggesting that the presence of the statine or Ahmppa unit could enhance the HIV-1 protease inhibitory activity. In addition, all of the compounds were not active at toward Hela, HepG2, and U2OS tumor cells at 100 μM in *in vitro* tests.

**Table 3:** Inhibitory activities of 1–9 against HIV-1 protease

Compd	IC_50_ (nM)	Compd	IC_50_ (nM)
1	1.79	6	9.90
2	3190.0	7	203.30
3	>10 000	8	138.60
4	92.10	9	782.10
5	22.27	Indinavir	1.82

## Experimental

### General experimental methods

ORs were measured using a Rudolph Research Autopol III automatic polarimeter. UV, CD, and IR spectra were recorded using a Cary 300 spectrometer, a JASCO J-815 CD spectrometer, and a Nicolet 5700 FT-IR spectrometer (FT-IR microscope transmission), respectively. ^1^H and ^13^C NMR spectra were obtained at 600 and 150 MHz, respectively, using a Bruker-AVIIIHD-600 spectrometer with solvent peaks used as references. HR-ESIMS data were measured using a Micromass Autospec-Ultima ETOF spectrometer and Thermo LTQ Orbitrap XL mass spectrometer. Column chromatography was performed using silica gel (200–300 mesh, Qingdao Marine Chemical Inc., China) and MCI gel CHP20P (Mitsubishi Chemical Corporation, Tokyo, Japan). HPLC separation was performed using an Agilent 1200 series (quaternary pump, autosampler, diode array detector) with a Shiseido Capcell-Pak C_18_ MGII column (5 μm, 250 × 10 mm). TLC was performed using glass-precoated silica gel GF254 plates (Qingdao Marine Chemical Inc., Qingdao, China). Cervical cancer (Hela), hepatocellular carcinoma (HepG2), and human osteosarcoma (U2OS) cell lines were obtained from the National Infrastructure of Cell Line Resource (Beijing, China).

### Microorganism and fermentation

Strain CPCC 202950 was identified as a member of the genus *Streptomyces* on the basis of 16S rRNA sequence analysis and deposited at the China Pharmaceutical Culture Collection (Institute of Medicinal Biotechnology, Chinese Academy of Medical Sciences and Peking Union Medical College, no. CPCC 202950).

The strain was cultured on slants of YM (0.4% yeast extract, 1% malt extract, 0.4% glucose, and 1.2% agar) at 28 °C for 7 d. Agar plugs were cut into small pieces (about 0.5 × 0.5 × 0.5 cm^3^) under aseptic conditions and 5 of these pieces were used to inoculate in three Erlenmeyer flasks (500 ml), each containing 100 ml of media (glucose 0.5%, yeast extract 0.5%, soluble starch 2.0%, soybean meal 1%, peptone 0.5%, beef extract 0.5%, corn steep liquor 0.4%, CaCO_3_ 0.4%, and CoCl_2_·6H_2_O 0.002%), and the final pH was adjusted to 7.2. After sterilization, three flasks of the inoculated media were incubated at 28 °C on a rotary shaker at 220 rpm for two days to prepare the seed culture. Spore inoculum was prepared by suspending the seed culture in sterile, distilled H_2_O to give a final spore/cell suspension of 1 × 10^6^/ml. Fermentation was carried out in 30 Fernbach flasks (500 ml), each containing 80 g of rice. Distilled H_2_O (120 ml) was added to each flask, and the contents were soaked overnight before autoclaving at 15 psi for 30 min. After cooling to room temperature, each flask was inoculated with 10.0 ml of the spore inoculum and incubated at 28 °C for 30 day.

### Extraction and isolation

The fermented material was ultrasonicated with 95% EtOH (2 × 8.0 l × 40 min), and the EtOH extracts were combined and evaporated under reduced pressure to yield an aqueous suspension (2.0 l). The suspension was partitioned with EtOAc (4 × 2.0 l). The EtOAc extract (15.0 g) was chromatographed over a silica column using a gradient elution of increasing MeOH (0–100%) in CH_2_Cl_2_ to give 10 fractions (F_1_–F_10_). The fraction of F_6_ was also subjected to silica gel CC using a gradient elution of increasing MeOH (0–100%) in CH_2_Cl_2_ to give six parts (F_6-1_–F_6-6_). Further purification of F_6-1_ and F_6-3_ with reversed-phase semipreparative HPLC (Capcell PAK C_18_-MGII 5 μm, 10 mm × 250 mm, 1.5 ml min^−1^, 37% MeCN in 0.1% trifluoroacetic acid) yielded 2 (13.5 mg) and 1 (8.6 mg), respectively. The aqueous phase was chromatographed over MCI gel (CHP20P, 1 l) with successive elution using H_2_O, 10% EtOH, 20% EtOH, 35% EtOH, 50% EtOH, and 70% EtOH to afford fractions M_1_–M_5_. M_4_ was further separated by reversed-phase semipreparative HPLC (Capcell PAK C_18_-MGII 5 μm, 10 mm × 250 mm, 1.5 ml min^−1^, 35% MeCN in 0.1% trifluoroacetic acid) to afford 3 (102.0 mg).

### Physical–chemical properties of 1–3

#### Ahmpatinin ^i^Bu (1)

White amorphous powder; [*α*]^20^_D_ −75.0 (*c* 0.5, CH_3_CN : H_2_O 1 : 1); UV (MeOH) *λ*_max_ (log *ε*) 223 (4.44), 275 (3.56) nm; IR *ν*_max_ 3285, 2961, 2925, 2873, 2851, 1635, 1547, 1515, 1468, 1390, 1249, 1183, 1148, 1099, 1038, 722 cm^−1^; ^1^H NMR (DMSO-*d*_6_, 600 MHz) data and ^13^C NMR (DMSO-*d*_6_, 150 MHz) data, see Table 1. (+)-HR-ESIMS *m*/*z* 736.4474 [M + H]^+^ (calcd for C_37_H_62_N_5_O_10_, 736.4439).

#### Statinin ^i^Bu (2)

White amorphous powder; [*α*]^20^_D_ −85.5 (*c* 0.6, CH_3_CN : H_2_O 1 : 1); IR *ν*_max_ 3285, 3080, 2964, 2874, 1727, 1636, 1545, 1466, 1389, 1284, 1229, 1150, 1098, 1053, 721 cm^−1^; ^1^H NMR (DMSO-*d*_6_, 600 MHz) data and ^13^C NMR (DMSO-*d*_6_, 150 MHz) data, see Table 1. (−)-HR-ESIMS *m*/*z* 513.3275 [M − H]^−^ (calcd for C_25_H_45_N_4_O_7_, 513.3283).

#### (−)-(*S*)-2-[3-(6-Methylheptanamido)-2-oxopyrrolidin-1-yl]acetic acid (3)

White amorphous powder; [*α*]^20^_D_ −100.5 (*c* 1.0, MeCN); CD (MeCN) 216 (Δ*ε* −15.88) nm; IR *ν*_max_ 3287, 3066, 2947, 2871, 1700, 1644, 1551, 1465, 1439, 1397, 1300, 1263, 1188, 1148, 926, 721, 636 cm^−1^; ^1^H NMR (DMSO-*d*_6_, 600 MHz) data and ^13^C NMR (DMSO-*d*_6_, 150 MHz) data, see Table 1. (+)-HR-ESIMS *m*/*z* 285.1802 [M + H]^+^ (calcd for C_14_H_25_N_2_O_4_, 285.1808).

#### Statine^18^

The (3*S*,4*S*)- and (3*R*,4*S*)-isomers of statine were prepared from Boc-l-Leu following the published procedure to prepare the mixture of statine: ^1^H NMR (DMSO-*d*_6_, 600 MHz) (3*S*,4*S*)-isomer (major product) *δ* 0.87 (6H, d, *J* = 6.6 Hz, ζ-CH_3_ and ζ′-CH_3_), 1.31 (1H, m, δ-H_a_), 1.45 (1H, m, δ-H_b_), 1.69 (1H, m, ε-H), 2.43 (1H, dd, *J* = 8.4, 15.6 Hz, α-H_a_), 2.55 (1H, dd, *J* = 4.8, 15.6 Hz, α-H_b_), 3.09 (1H, m, β-H), 3.93 (1H, m, γ-H), 5.70 (1H, br s, β-OH), 7.62 (2H, br s, γ-NH_2_), 12.32 (1H, br s, –COOH̲); (3*R*,4*S*)-isomer (minor product) *δ* 0.84 (3H, d, *J* = 6.6 Hz, ζ-CH_3_), 0.89 (3H, d, *J* = 6.6 Hz, ζ′-CH_3_), 1.27 (1H, m, δ-H_a_), 1.40 (1H, m, δ-H_b_), 1.64 (1H, m, ε-H), 2.29 (1H, dd, *J* = 8.4, 15.6 Hz, α-H_a_), 2.43 (1H, dd, *J* = 4.8, 15.6 Hz, α-H_b_), 3.13 (1H, m, β-H), 4.11 (1H, m, γ-H), 5.50 (1H, br s, β-OH), 7.74 (2H, br s, γ-NH_2_), 12.32 (1H, br s, –COOH̲).

#### Ahmppa^18^

The (3*S*,4*S*)- and (3*R*,4*S*)-isomers along with the (3*R*,4*R*)- and (3*S*,4*R*)-isomers of Ahmppa were prepared from *N*-Boc-4-methoxy-l-Tyr and *N*-Boc-4-methoxy-d-Tyr following the procedure to prepare the mixture of statine isomers, respectively. The (3*S*,4*S*)- and (3*R*,4*S*)-isomers and (3*R*,4*R*)- and (3*S*,4*R*)-isomers of the Ahmppa mixture were subjected to reversed-phase semipreparative HPLC (Capcell PAK C_18_-MGII 5 μm, 10 mm × 250 mm, 1.5 ml min^−1^, 20% MeCN in 0.1% trifluoroacetic acid) to yield (3*S*,4*S*)- and (3*R*,4*S*)-Ahmppa and (3*R*,4*R*)- and (3*S*,4*R*)-Ahmppa, respectively. ^1^H NMR (DMSO-*d*_6_, 600 MHz) (3*S*,4*S*) and (3*R*,4*R*)-isomer (major product) *δ* 2.46 (2H, br d, *J* = 6.0 Hz, α-H_2_), 2.81 (2H, br d, *J* = 7.2 Hz, δ-H_2_), 3.26 (1H, m, β-H), 3.73 (3H, s, 4-OCH_3_), 3.87 (1H, m, γ-H), 6.89 (2H, d, *J* = 8.4 Hz, H-2 and H-6), 7.20 (2H, d, *J* = 8.4 Hz, H-3 and H-5), 5.78 (1H, br s, β-OH), 7.92 (2H, br s, γ-NH_2_), 12.23 (1H, br s, –COOH̲); (3*R*,4*S*) and (3*S*,4*R*)-isomer (minor product) *δ* 2.29 (1H, dd, *J* = 9.0, 15.6 Hz, α-H_a_), 2.54 (1H, dd, *J* = 3.6, 15.6 Hz, α-H_b_), 2.70 (1H, dd, *J* = 7.8, 14.4 Hz, δ-H_a_), 2.80 (1H, dd, *J* = 6.6, 14.4 Hz, δ-H_b_), 3.38 (1H, m, β-H), 3.73 (3H, s, 4-OCH_3_), 4.10 (1H, m, γ-H), 6.89 (2H, d, *J* = 8.4 Hz, H-2 and H-6), 7.19 (2H, d, *J* = 8.4 Hz, H-3 and H-5), 5.56 (1H, br s, β-OH), 7.80 (2H, br s, γ-NH_2_), 12.31 (1H, br s, –COOH̲).

#### (3*S*,4*S*)-Statine from acid hydrolysates of 1

Approximately 5.0 mg of 1 was hydrolyzed with 2 ml of 6 M HCl at 110 °C for 16 h. The hydrolysate was then evaporated to dryness and redissolved in H_2_O. The solution was chromatographed by reversed-phase semipreparative HPLC (Capcell PAK C_18_-AQ 5 μm, 10 mm × 250 mm, 1.5 ml min^−1^, 14% MeCN in 0.1% trifluoroacetic acid) to yield (3*S*,4*S*)-statine (0.5 mg): [*α*]^20^_D_ = −19.5 (*c* 0.05, H_2_O); ^1^H NMR (DMSO-*d*_6_, 600 MHz) *δ* 0.87 (6H, d, *J* = 6.6 Hz, ζ-CH_3_ and ζ′-CH_3_), 1.32 (1H, m, δ-H_a_), 1.45 (1H, m, δ-H_b_), 1.69 (1H, m, ε-H), 2.43 (1H, dd, *J* = 7.8, 15.6 Hz, α-H_a_), 2.56 (1H, dd, *J* = 4.8, 15.6 Hz, α-H_b_), 3.09 (1H, m, β-H), 3.93 (1H, m, γ-H), 5.70 (1H, br s, β-OH), 7.63 (2H, br s, γ-NH_2_), 12.33 (1H, br s, –COOH̲).

#### Chiral-phase HPLC analysis of the acid hydrolysate of 1^29,30^

Ahmpatinin ^i^Bu (1, 0.5 mg) was dissolved in 6 N HCl (1 ml) and heated at 110 °C for 16 h. After cooling to room temperature (rt), the solvent was evaporated, and traces of HCl were removed by repeated drying under vacuum with distilled H_2_O. The dried hydrolysate was dissolved in 200 μl of 2 mM CuSO_4_/H_2_O solution. The hydrolysate of 1 and authentic (3*S*,4*S*)-, (3*R*,4*S*)-, (3*R*,4*R*)-, and (3*S*,4*R*)-Ahmppa were analyzed with a chiral-phase column (MCI GEL CRS10W, 4.6 × 50 mm, Mitsubishi Chemical Corporation) using 2 mM CuSO_4_/H_2_O solution as the mobile phase with flow rate at 0.8 ml min^−1^ and UV detection at 254 nm on a Shimadzu LC-20A HPLC instrument. Ahmppa residue (*t*_R_ = 12.07 min) in ahmpatinin ^i^Bu (1) was found to correspond to the (3*S*,4*S*)-configuration by comparison of the retention times (*t*_R_, min) with those of standard Ahmppa units: (3*S*,4*S*)-Ahmppa (11.96), (3*R*,4*S*)-Ahmppa (10.63), (3*R*,4*R*)-Ahmppa (10.06), and (3*S*,4*R*)-Ahmppa (10.36).

#### HPLC analysis of the acid hydrolysates of 1 and 2 using Marfey's method

Approximately 0.5 mg of 1 and 2 were hydrolyzed with 1 ml of 6 N HCl at 110 °C for 16 h. The hydrolysate was evaporated to dryness and redissolved in H_2_O (200 μl). To one portion (100 μl), 100 μl of a 1% solution of 1-fluoro-2,4-dinitrophenyl-5-l-alanine amide (FDAA) in acetone and 20 μl of 1 M NaHCO_3_ were added. The reaction mixture was heated at 40 °C for 1 h, cooled to room temperature, neutralized with 2 M HCl (10 μl), and diluted with MeCN (100 μl). Similarly, the standard d-Ala, l-Ala, d-Leu, and l-Leu were derivatized separately. The Marfey's derivatives of the hydrolysate and standards were analyzed by HPLC using the following conditions. Column, Agilent ZORBAX SB-Aq C18 column (5 μm, 4.6 mm × 150 mm); flow rate, 1.0 ml min^−1^; solvent A, 0.1% trifluoroacetic acid in an aqueous solution; solvent B, MeCN; elution, 20–50% B in A over 60 min; UV detection at 340 nm; column temperature, 30 °C. The retention times for the FDAA derivatives of the standards d-Ala, l-Ala, d-Leu, and l-Leu were 39.93, 36.26, 49.43, and 44.10 min, respectively. The FDAA derivatives of the amino acids from the hydrolysate of 1 and 2 showed peaks at 36.30 (l-Ala) and 44.16 (l-Leu) min, respectively, and the amino acids were assigned to be l-Ala and l-Leu in 1 and 2, respectively.

#### ECD and OR calculations of compound 3

Conformational analysis of the *S*-enantiomer was carried out *via* Monte Carlo searching with the MMFF94s molecular mechanics force field using the Spartan 10 software.^31^ Seventy-two geometries having relative energies within 6 kcal mol^−1^ were optimized using DFT at the B3LYP/6-31G (d) level in vacuum with the Gaussian 09 program.^32^ The 52 B3LYP/6-31G(d)-optimized conformers with relative energies ranging from 0 to 4.0 kcal mol^−1^ were then reoptimized at the wB97XD/DGDZVP level in acetonitrile. ECD and OR computations for all wB97XD/DGDZVP-optimized conformers were carried out at the CAM-B3LYP/DGDZVP and B3LYP/DGDZVP levels in acetonitrile, respectively. Boltzmann statistics were computed for ECD simulations with a standard deviation of *σ* 0.3 eV. The ECD spectra were then simulated using the GaussSum 2.25 program.^33^ The final ECD spectrum of (*S*)-3 was obtained according to the Boltzmann distribution theory and the relative Gibbs free energy (Δ*G*).

#### Analysis of the inhibition of HIV-1 protease activity

This was measured using the previously described protocol.^15^ The positive control indinavir gave an IC_50_ value of 1.82 nM.

#### Cytotoxic activity assay

This was measured using the previously described protocol.^34^ The positive control doxorubicin showed *in vitro* cytotoxicity against cervical cancer (Hela), hepatocellular carcinoma (HepG2), and human osteosarcoma (U2OS) cell lines with IC_50_ values of 1.42, 1.56, and 2.78 μM, respectively.

## Conclusions

In summary, we have isolated two new linear peptides, ahmpatinin ^i^Bu (1) and statinin ^i^Bu (2), and a novel pyrrolidine anolog, (−)-(*S*)-2-[3-(6-methylheptanamido)-2-oxopyrrolidin-1-yl]acetic acid (3), from a *Streptomyces* sp. strain. The unusual amino acid, 4-amino-3-hydroxy-5-(4-methoxyphenyl)pentanoic acid (Ahmppa) of ahmpatinin ^i^Bu was identified for the first time from a natural source, and 3 represents the first reported instance of a natural pyrrolidine possessing a 2-(3-amino-2-oxopyrrolidin-1-yl)acetic acid system. In addition, ahmpatinin ^i^Bu (1) displayed significant inhibitory activity against HIV-1 protease.

## Conflicts of interest

There are no conflicts to declare.

## Supplementary Material

RA-008-C7RA13241G-s001
