# Supplementary material for: Ahmpatinin iBu, a new HIV-1 protease inhibitor, from Streptomyces sp. CPCC 202950
Source: RSC Adv. 2018 Jan 30;8(10):5138–44. doi: 10.1039/c7ra13241g (PMC9078123; doi:10.1039/c7ra13241g)
Supplement: RA-008-C7RA13241G-s001 [file RA-008-C7RA13241G-s001.pdf]

Electronic Supplementary Material (ESI) for RSC Advances.
This journal is © The Royal Society of Chemistry 2018

Ahmpatinin ^{i}Bu, a new HIV-1 protease inhibitor, from a *Streptomyces* sp. CPCC 202950

Ming-Hua Chen,‡[ac] Shan-Shan Chang,‡[ab] Biao Dong, [a] Li-Yan Yu,[a] Ye-Xiang Wu,[a] Ren-Zhong Wang,[a] Wei Jiang,[a] Zeng-Ping Gao,[*b] Shu-Yi Si [*a]

[a.] *Institute of Medicinal Biotechnology, Chinese Academy of Medical Sciences & Peking Union Medical College, Beijing 100050, China. E-mail: sisyimb@hotmail.com (S. Si)*

[b.] *School of Chinese Materia Medica, Beijing University of Chinese Medicine, Beijing 100102, China. E-mail: gaozp@bucm.edu.cn (Z. Gao)*

[c.] *Key Laboratory for Uighur Medicine, Institute of Materia Medica of Xinjiang, Urumqi 830004, China.*

Supporting Information

List of Content

No.	Content		Page
1	**Figure S1.**	The IR Spectrum of Compound **1**	S3
2	**Figure S2.**	The (+)-HRESIMS Spectroscopic Data of Compound **1**	S4
3	**Figure S3.**	The ^{1}H NMR Spectrum of Compound **1** in DMSO–d_6	S5
4	**Figure S4.**	The ^{13}C NMR Spectrum of Compound **1** in DMSO–d_6	S6
5	**Figure S5.**	The DEPT Spectrum of Compound **1** in DMSO–d_6	S7
6	**Figure S6.**	The ^{1}H-^{1}H COSY Spectrum of Compound **1** in DMSO–d_6	S8
7	**Figure S7.**	The HSQC Spectrum of Compound **1** in DMSO–d_6	S9
8	**Figure S8.**	The HMBC Spectrum of Compound **1** in DMSO–d_6	S10
9	**Figure S9.**	The NOESY Spectrum of Compound **1** in DMSO–d_6	S11
10	**Figure S10.**	The IR Spectrum of Compound **2**	S12
11	**Figure S11.**	The (–)-HRESIMS Spectroscopic Data of Compound **2**	S13
12	**Figure S12.**	The ^{1}H NMR Spectrum of Compound **2** in DMSO–d_6	S14
13	**Figure S13.**	The ^{13}C NMR Spectrum of Compound **2** in DMSO–d_6	S15
14	**Figure S14.**	The DEPT Spectrum of Compound **2** in DMSO–d_6	S16
15	**Figure S15.**	The ^{1}H-^{1}H COSY Spectrum of Compound **2** in DMSO–d_6	S17
16	**Figure S16.**	The HSQC Spectrum of Compound **2** in DMSO–d_6	S18
17	**Figure S17.**	The HMBC Spectrum of Compound **2** in DMSO–d_6	S19
18	**Figure S18.**	The NOESY Spectrum of Compound **2** in DMSO–d_6	S20
19	**Figure S19.**	The IR Spectrum of Compound **3**	S21
20	**Figure S20.**	The (+)-HRESIMS Spectroscopic Data of Compound **3**	S22
21	**Figure S21.**	The ^{1}H NMR Spectrum of Compound **3** in DMSO–d_6	S23
22	**Figure S22.**	The ^{13}C NMR Spectrum of Compound **3** in DMSO–d_6	S24
23	**Figure S23.**	The DEPT Spectrum of Compound **3** in DMSO–d_6	S25
24	**Figure S24.**	The ^{1}H-^{1}H COSY Spectrum of Compound **3** in DMSO–d_6	S26
25	**Figure S25.**	The HSQC Spectrum of Compound **3** in DMSO–d_6	S27
26	**Figure S26.**	The HMBC Spectrum of Compound **3** in DMSO–d_6	S28
27	**Figure S27.**	Marfey's Analysis of Acid Hydrolysate of Compound **1**	S29
28	**Figure S28.**	Chiral-phase HPLC Analysis of Acid Hydrolysate of Compound **1**	S30

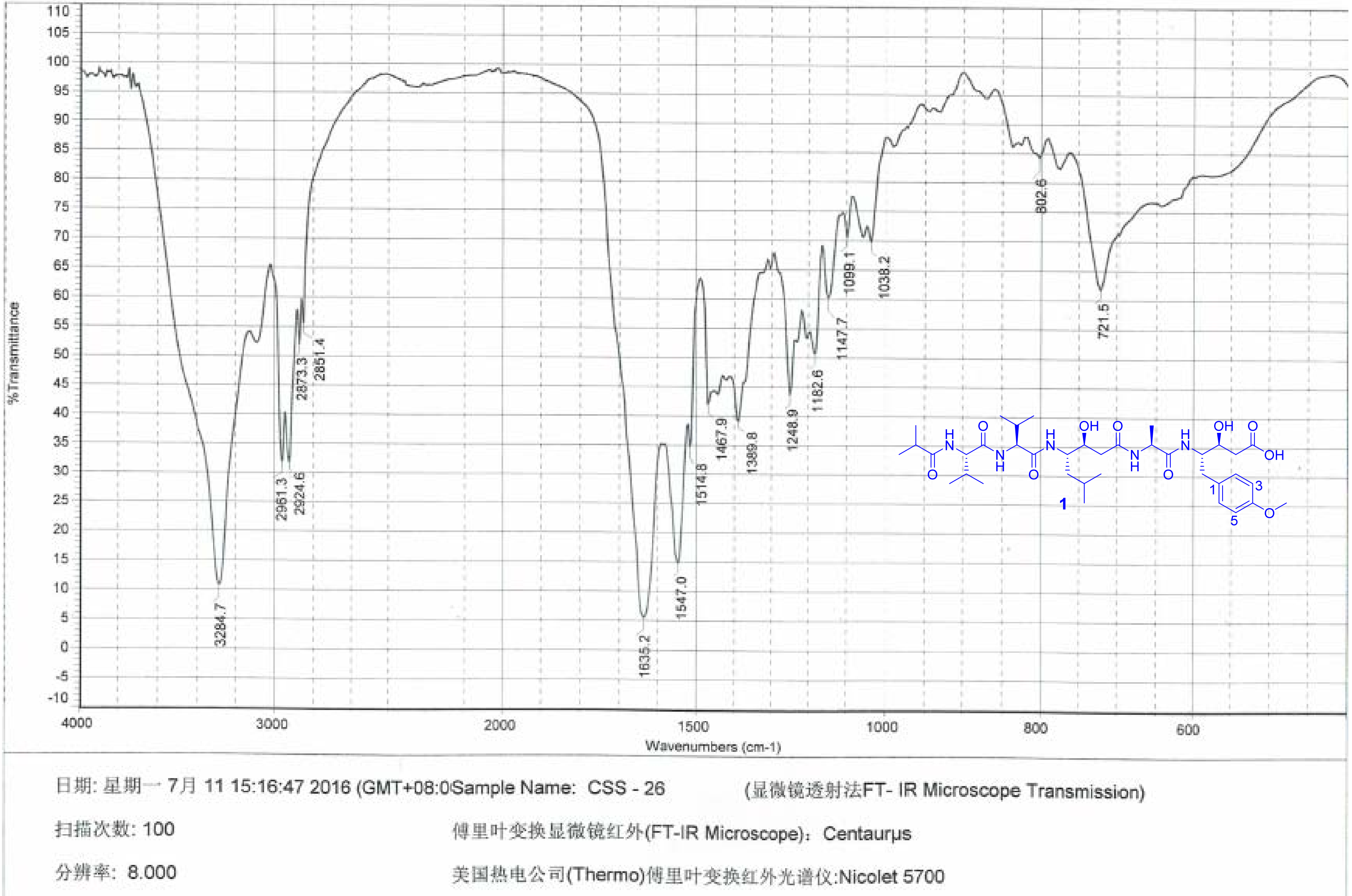

Figure S1. The IR Spectrum of Compound **1**

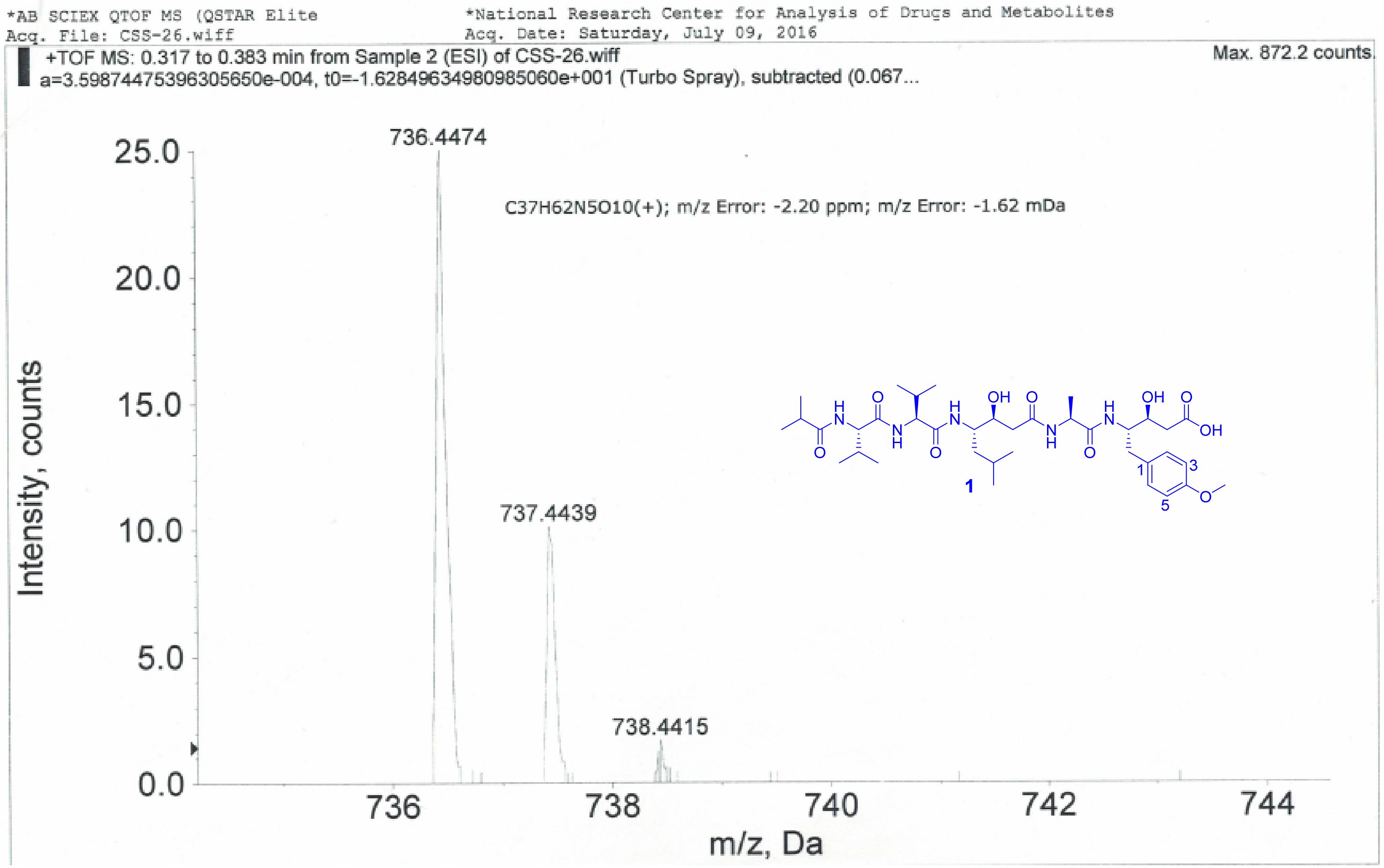

Figure S2. The (+)-HRESIMS Spectroscopic Data of Compound **1**

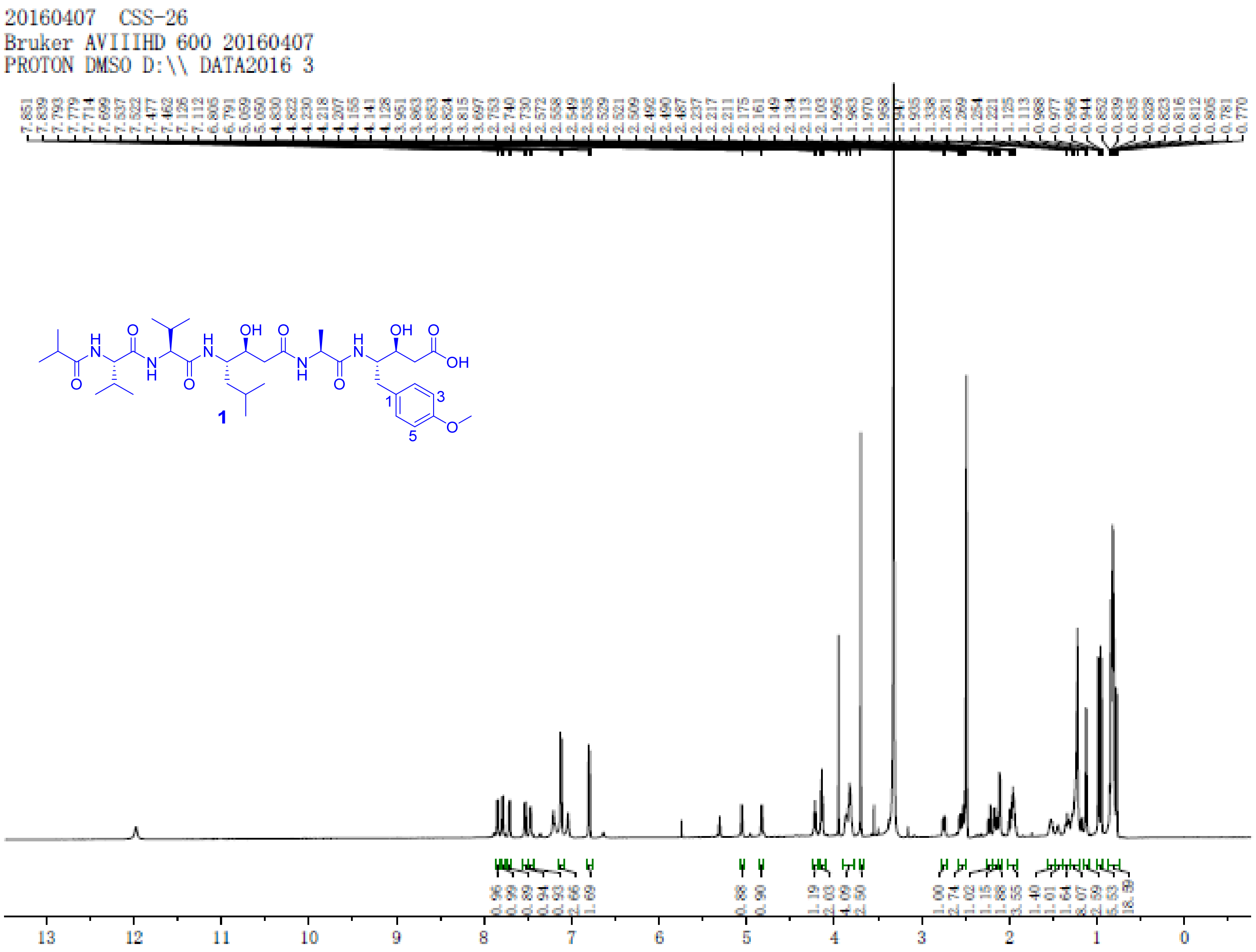

Figure S3. The ^{1}H NMR Spectrum of Compound **1** in DMSO−*d*6

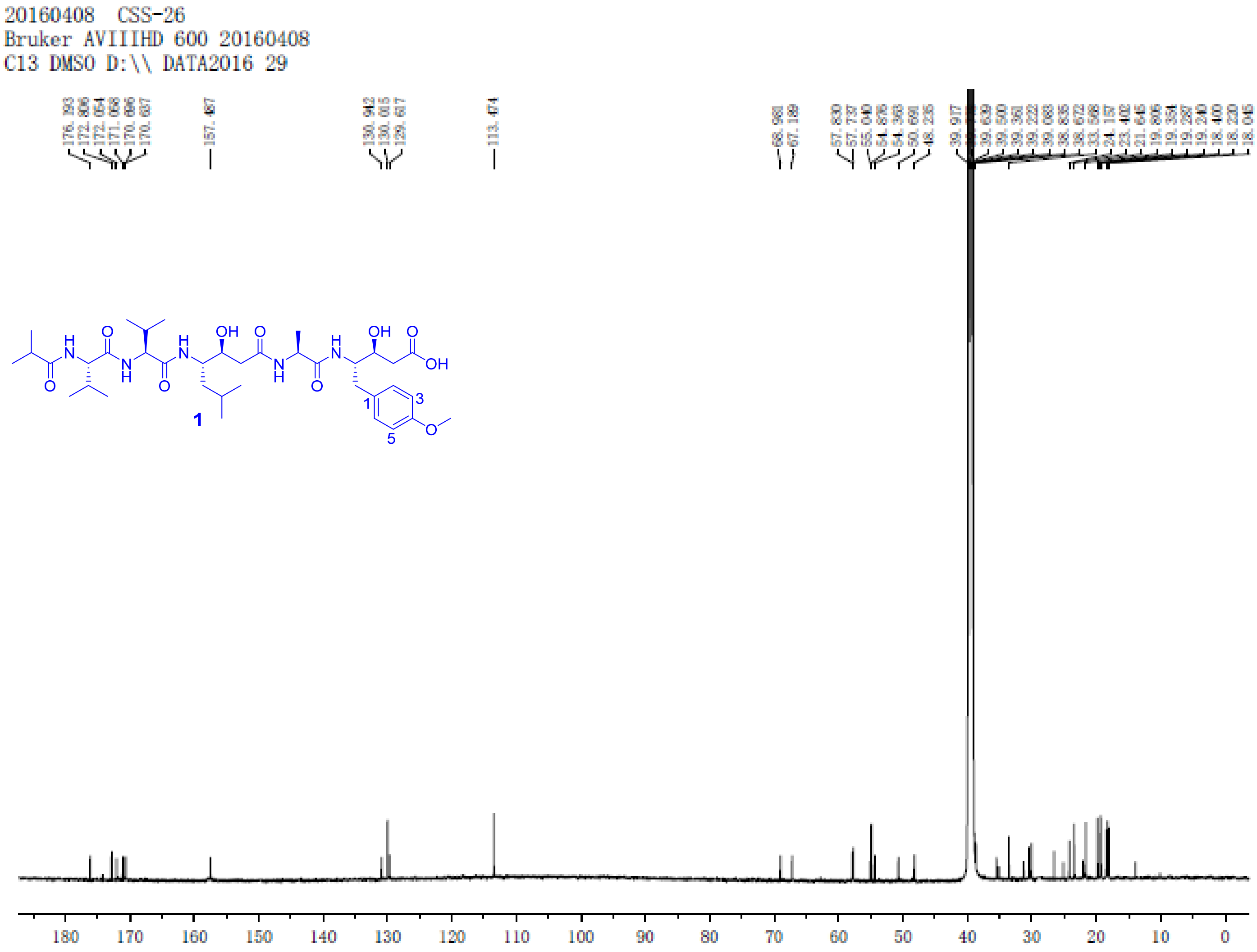

Figure S4. The ^{13}C NMR Spectrum of Compound **1** in DMSO–d_6

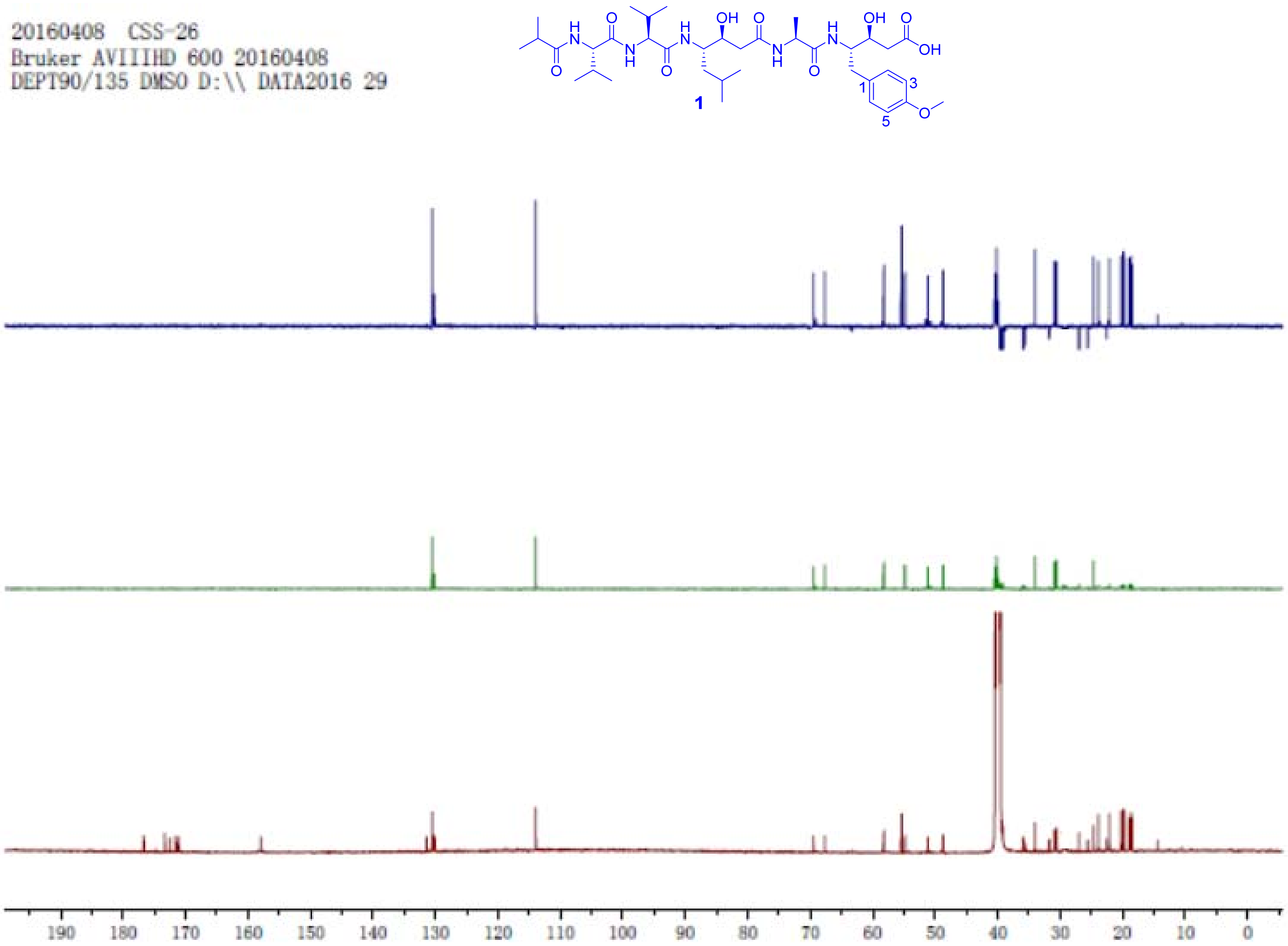

Figure S5. The DEPT Spectrum of Compound **1** in DMSO–d_6

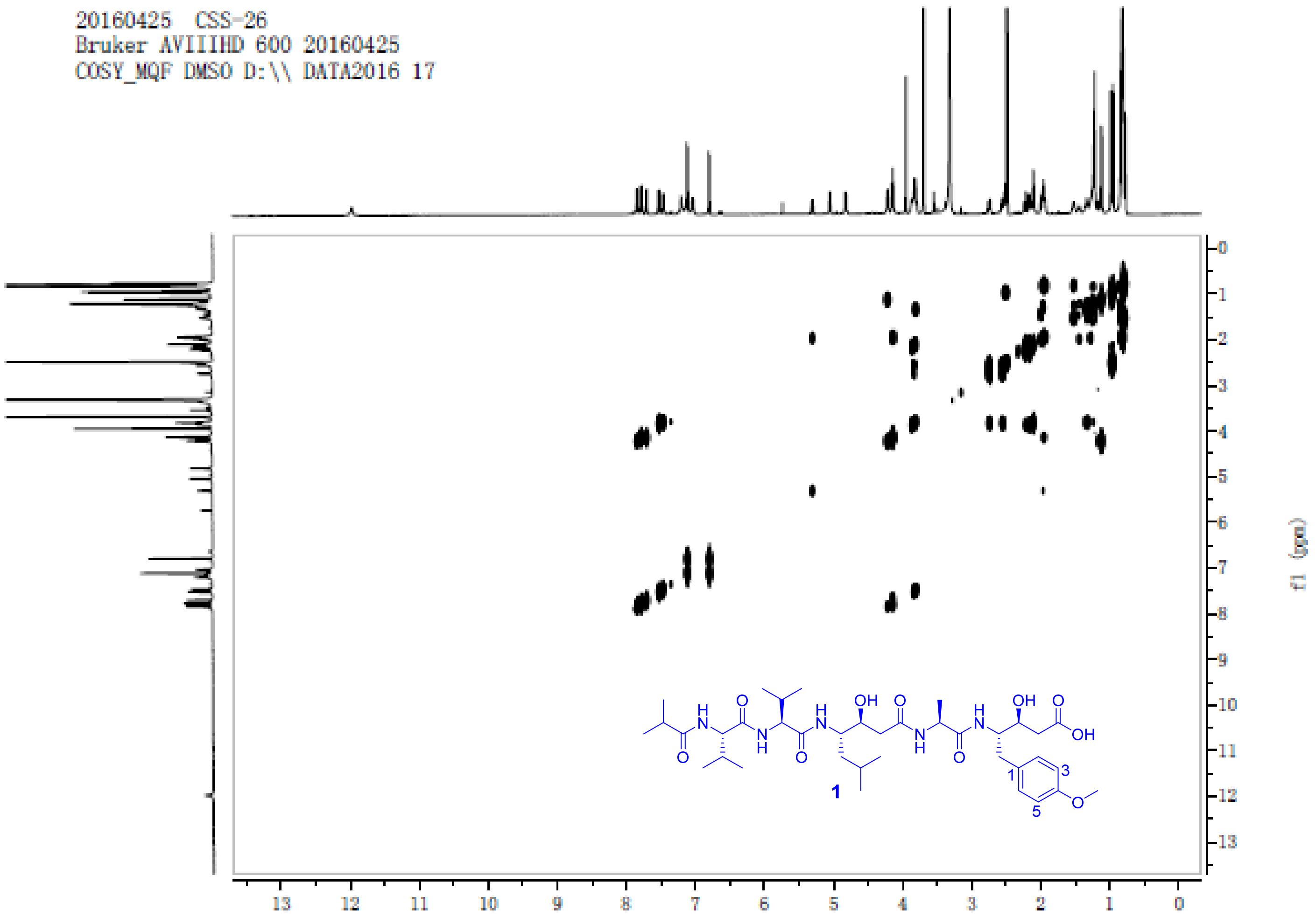

Figure S6. The ^{1}H-^{1}HCOSY Spectrum of Compound **1** in DMSO–d_6

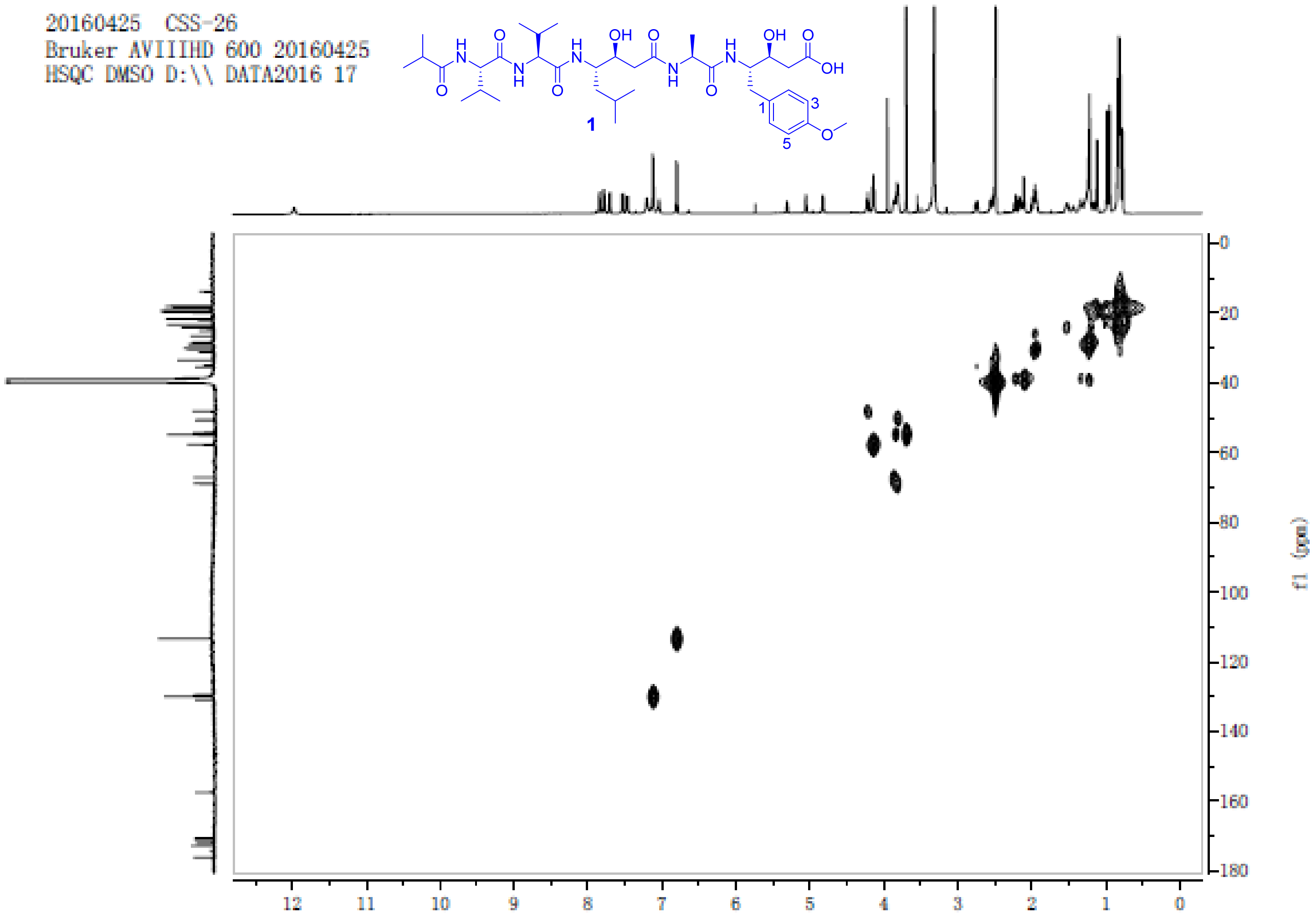

Figure S7. The HSQC Spectrum of Compound **1** in DMSO–*d*6

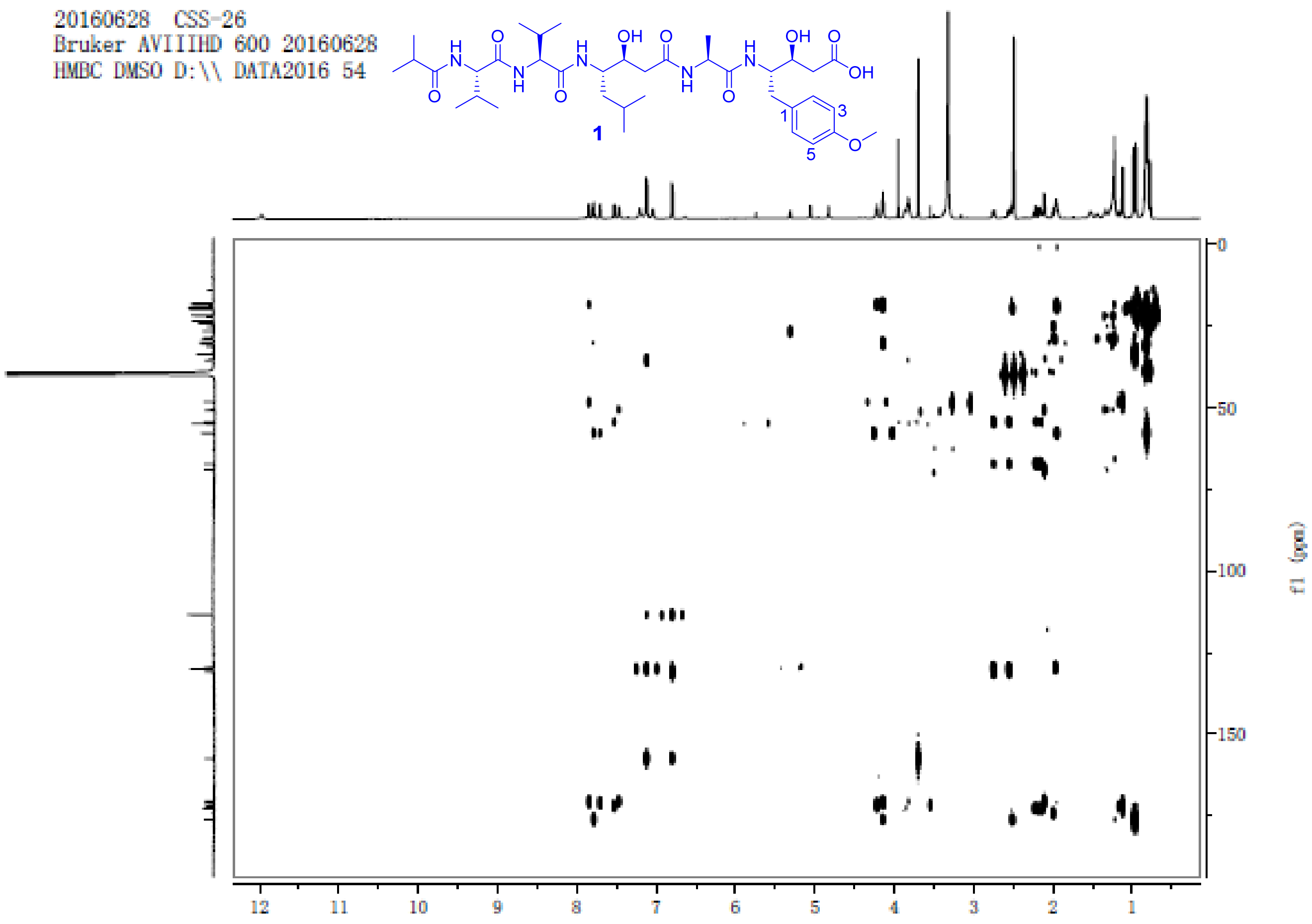

Figure S8. The HMBC Spectrum of Compound **1** in DMSO–d_6

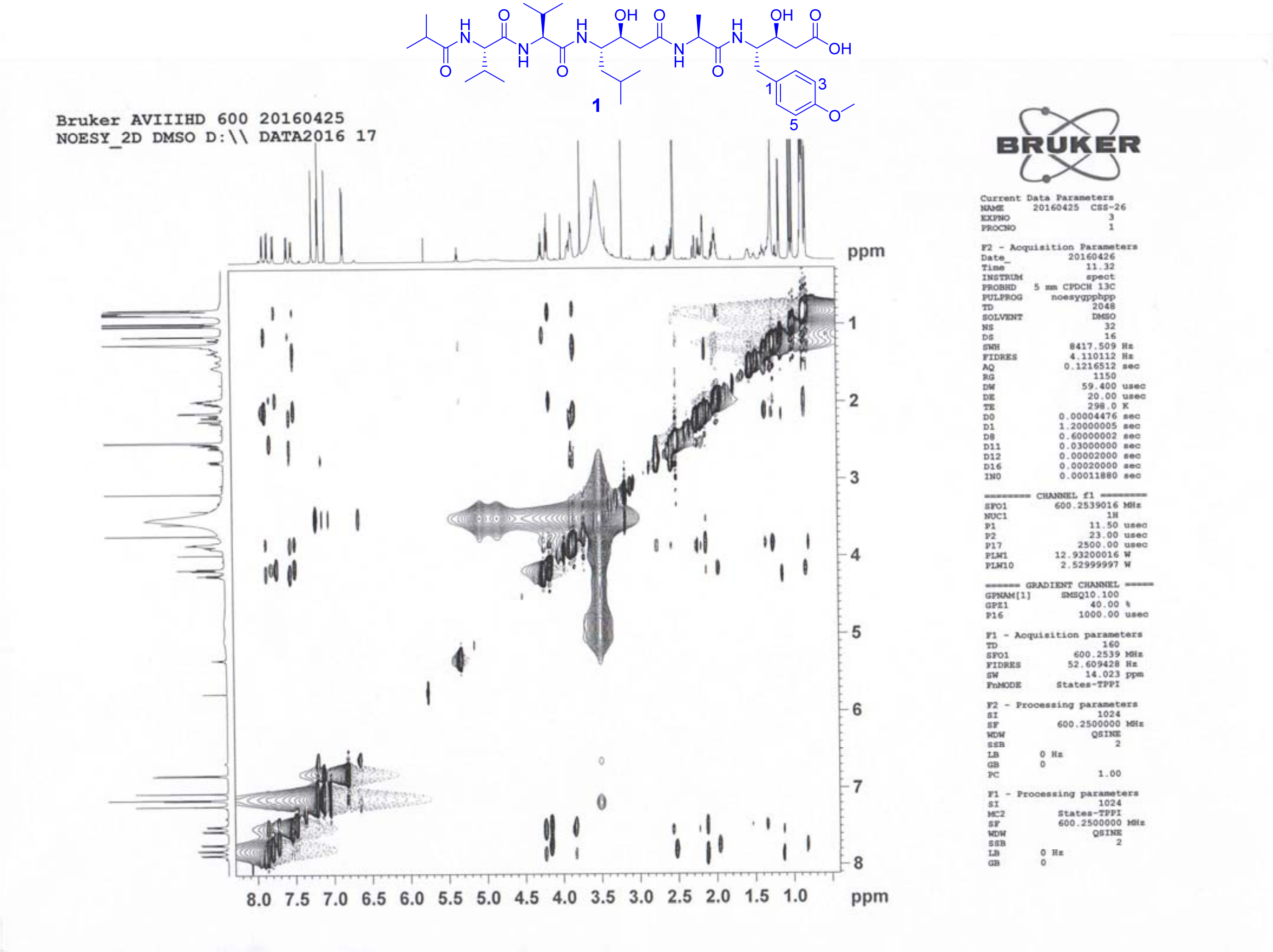

Figure S9. The NOESY Spectrum of Compound **1** in DMSO–d_6

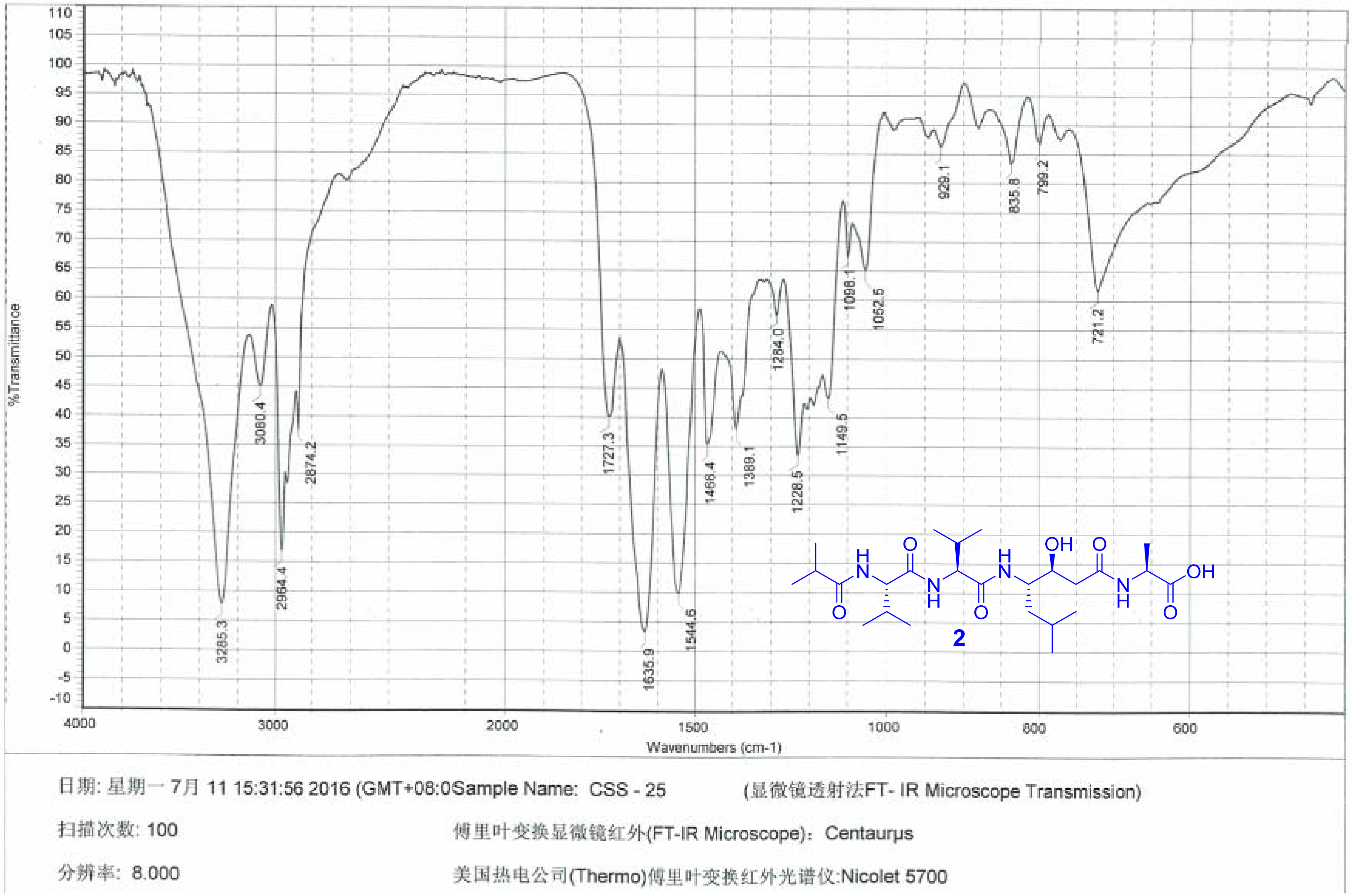

Figure S10. The IR Spectrum of Compound **2**

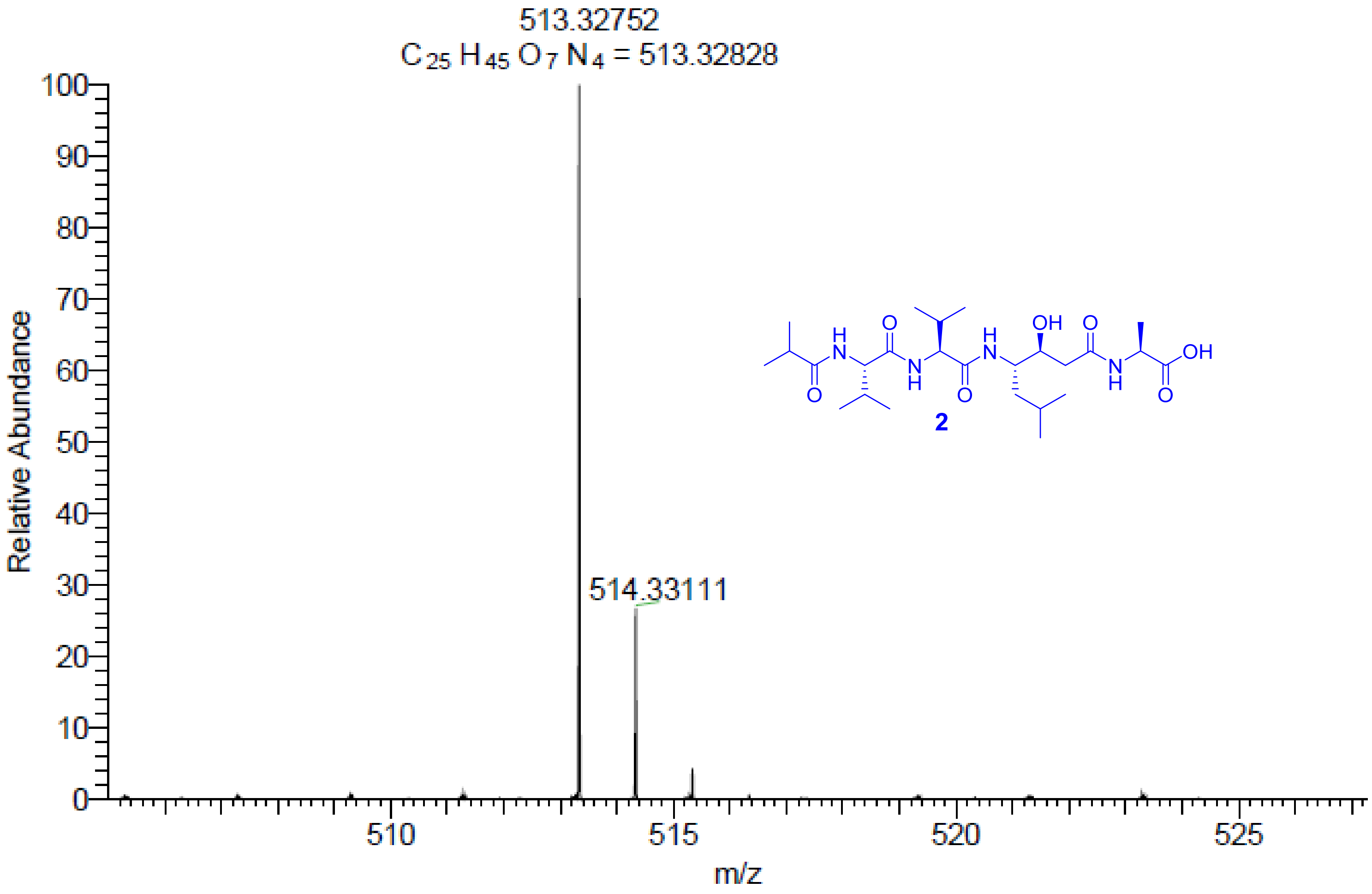

Figure S11. The (–)-HRESIMS Spectroscopic of Compound **2**

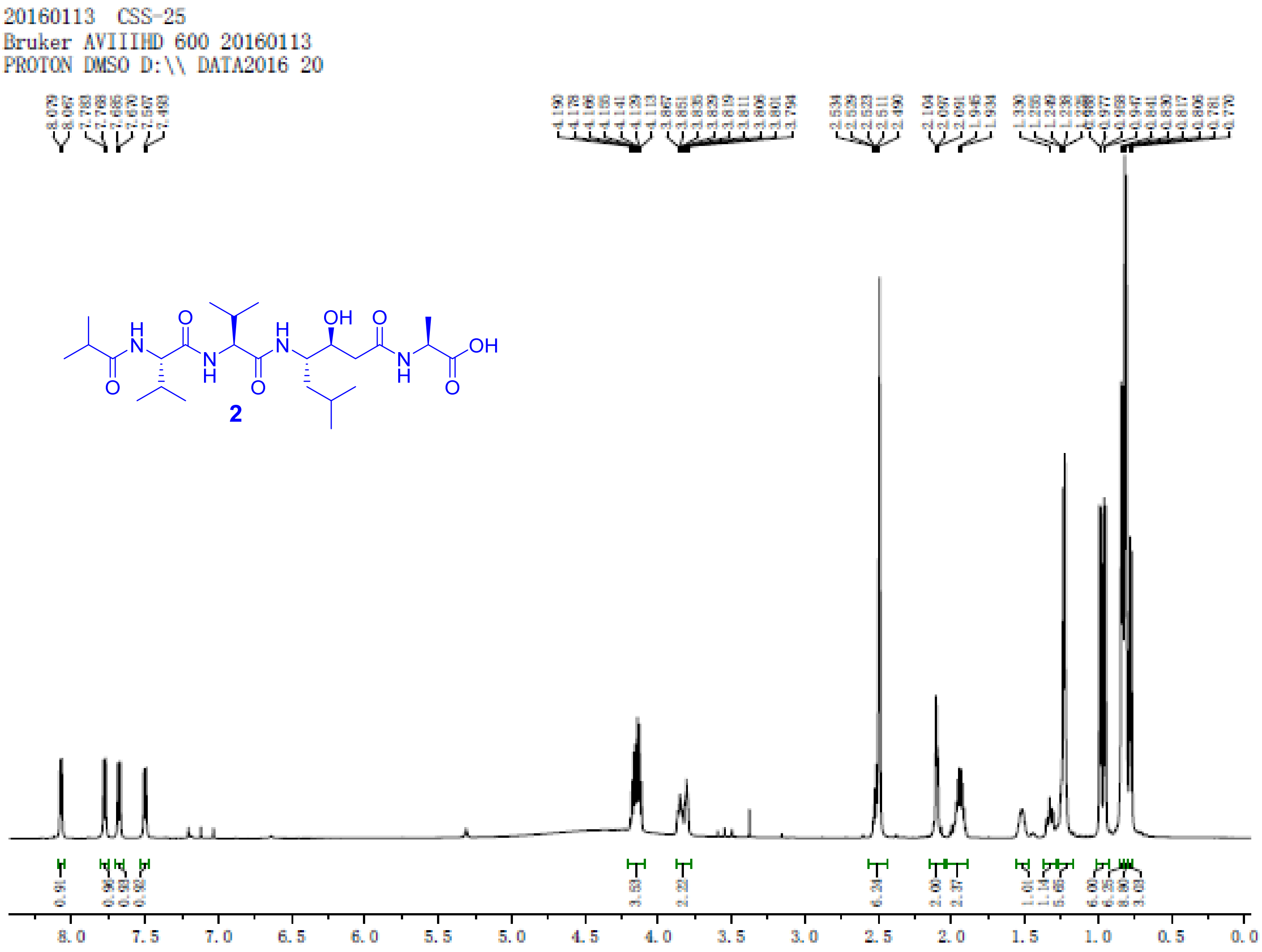

Figure S12. The ^{1}H NMR Spectrum of Compound **2** in DMSO–d_6

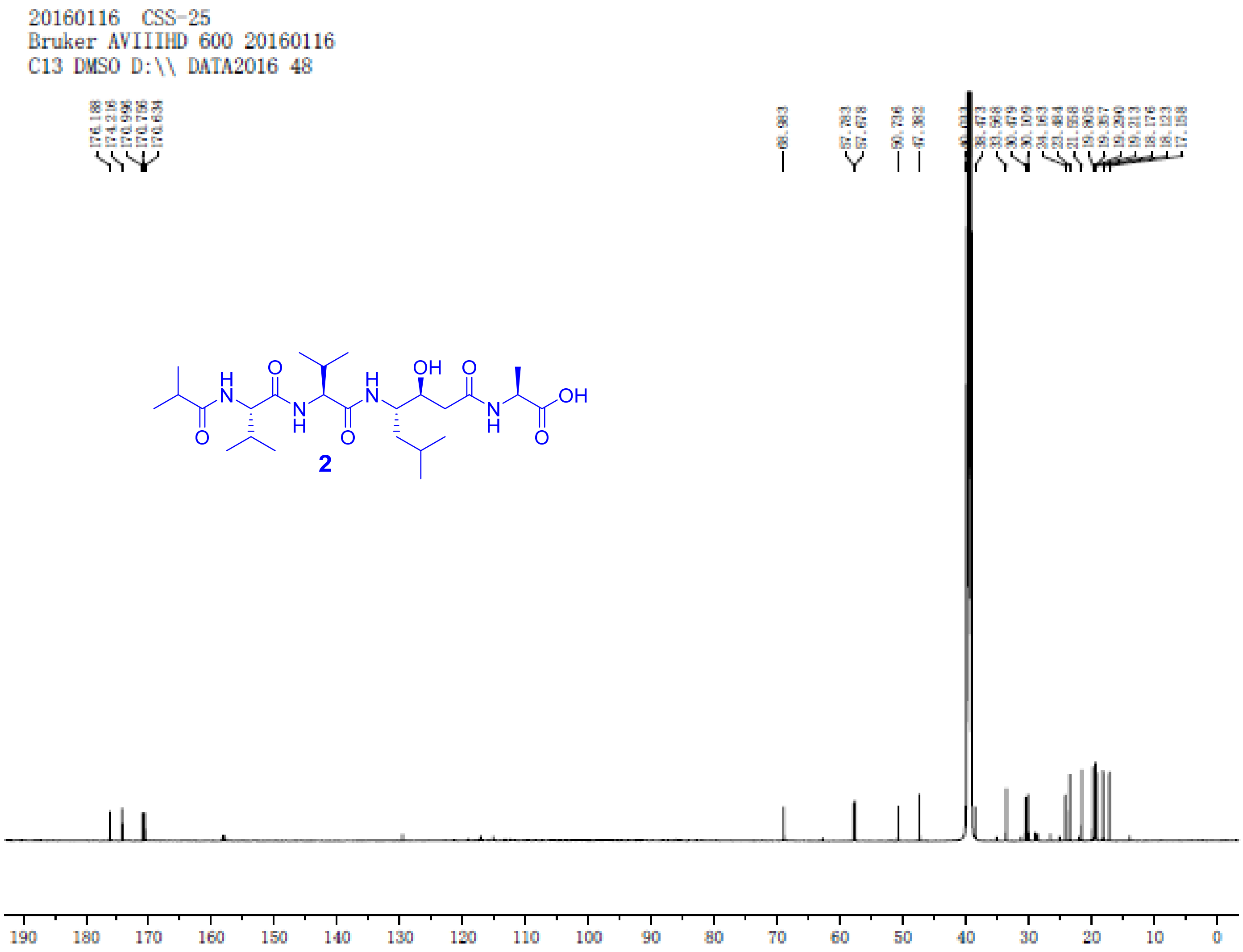

Figure S13. The ^{13}C NMR Spectrum of Compound **2** in DMSO–d_6

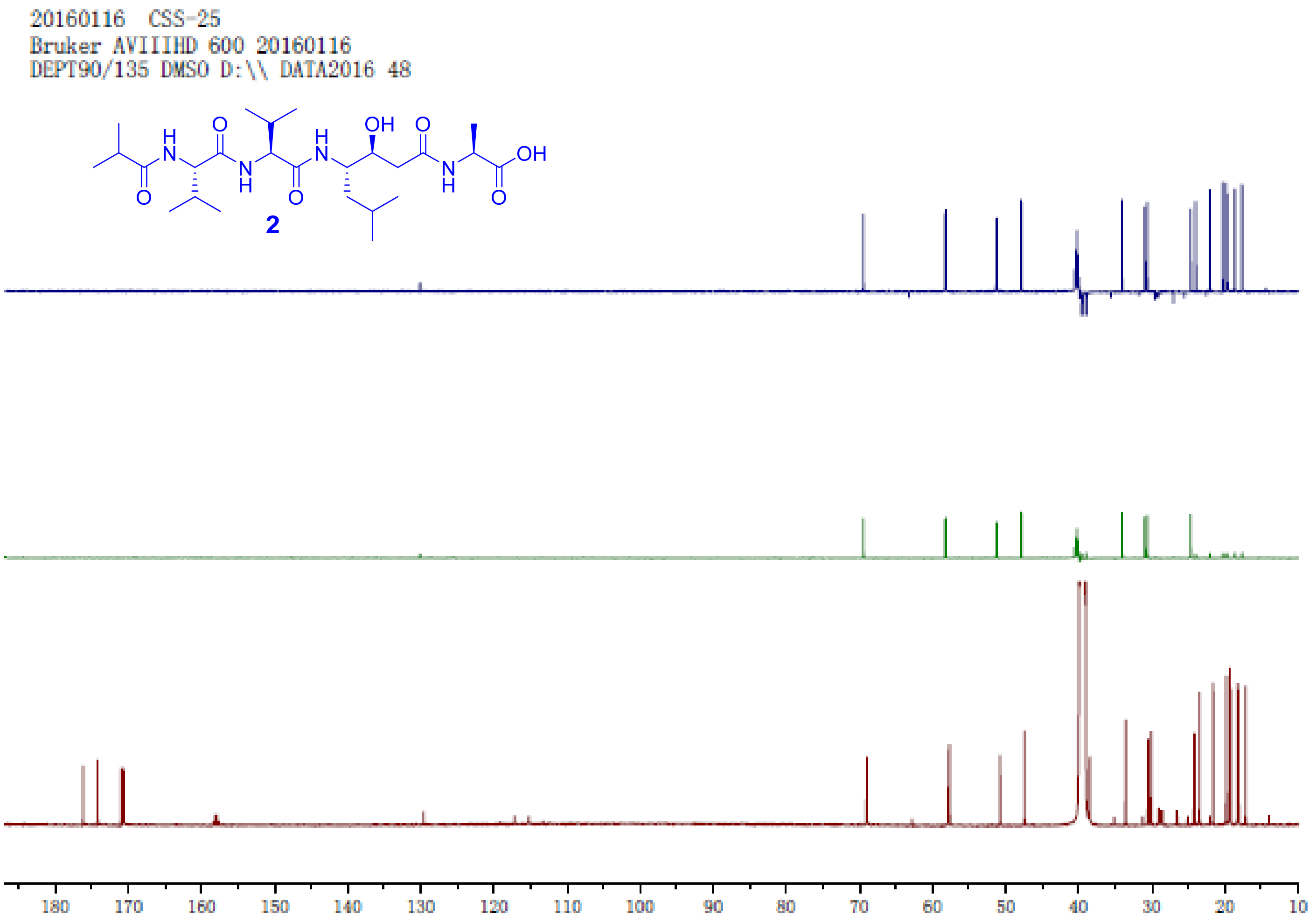

Figure S14. The DEPT Spectrum of Compound **2** in DMSO–d_6

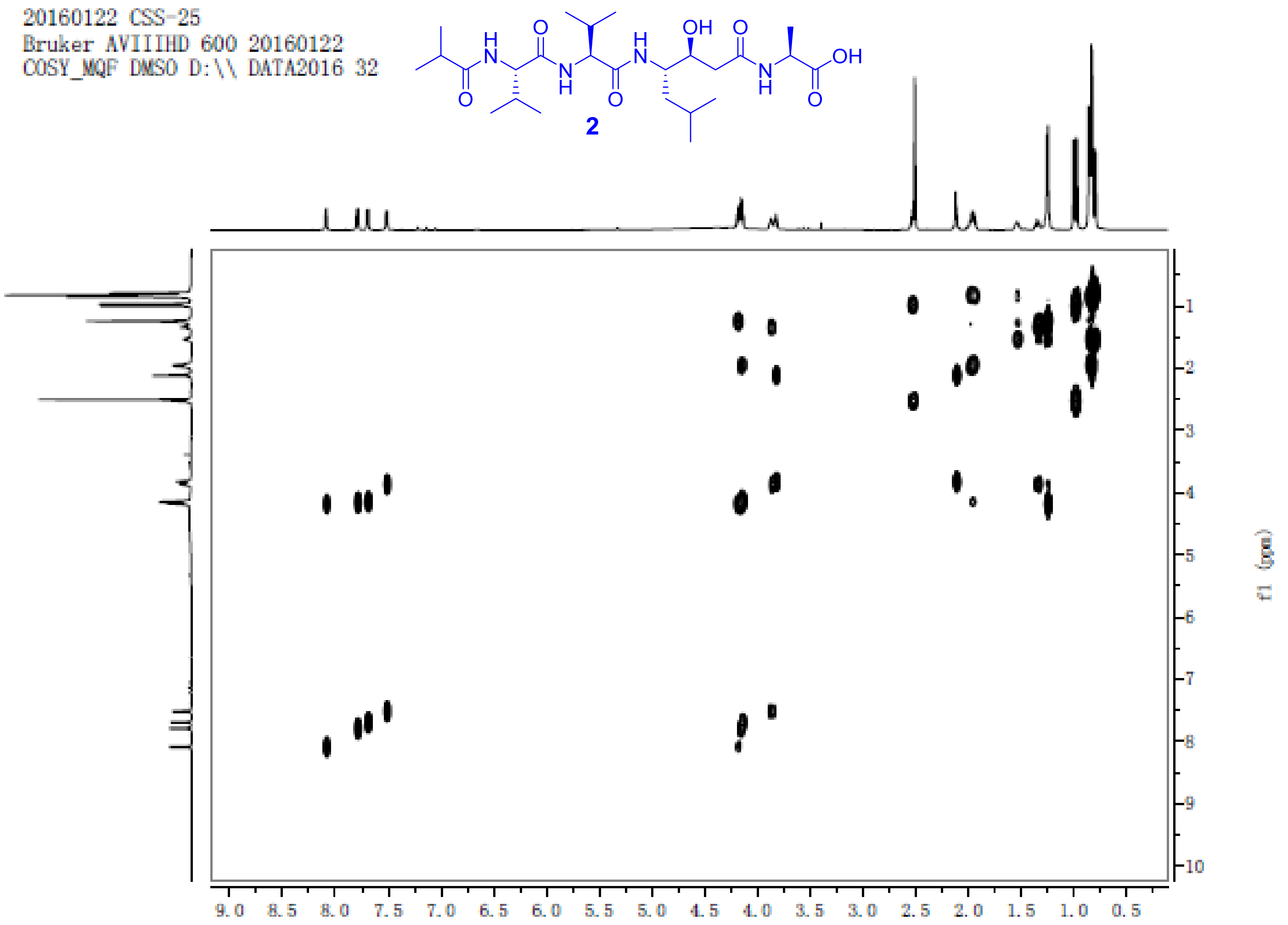

Figure S15. The ^{1}H-^{1}H COSY Spectrum of Compound **2** in DMSO–d_6

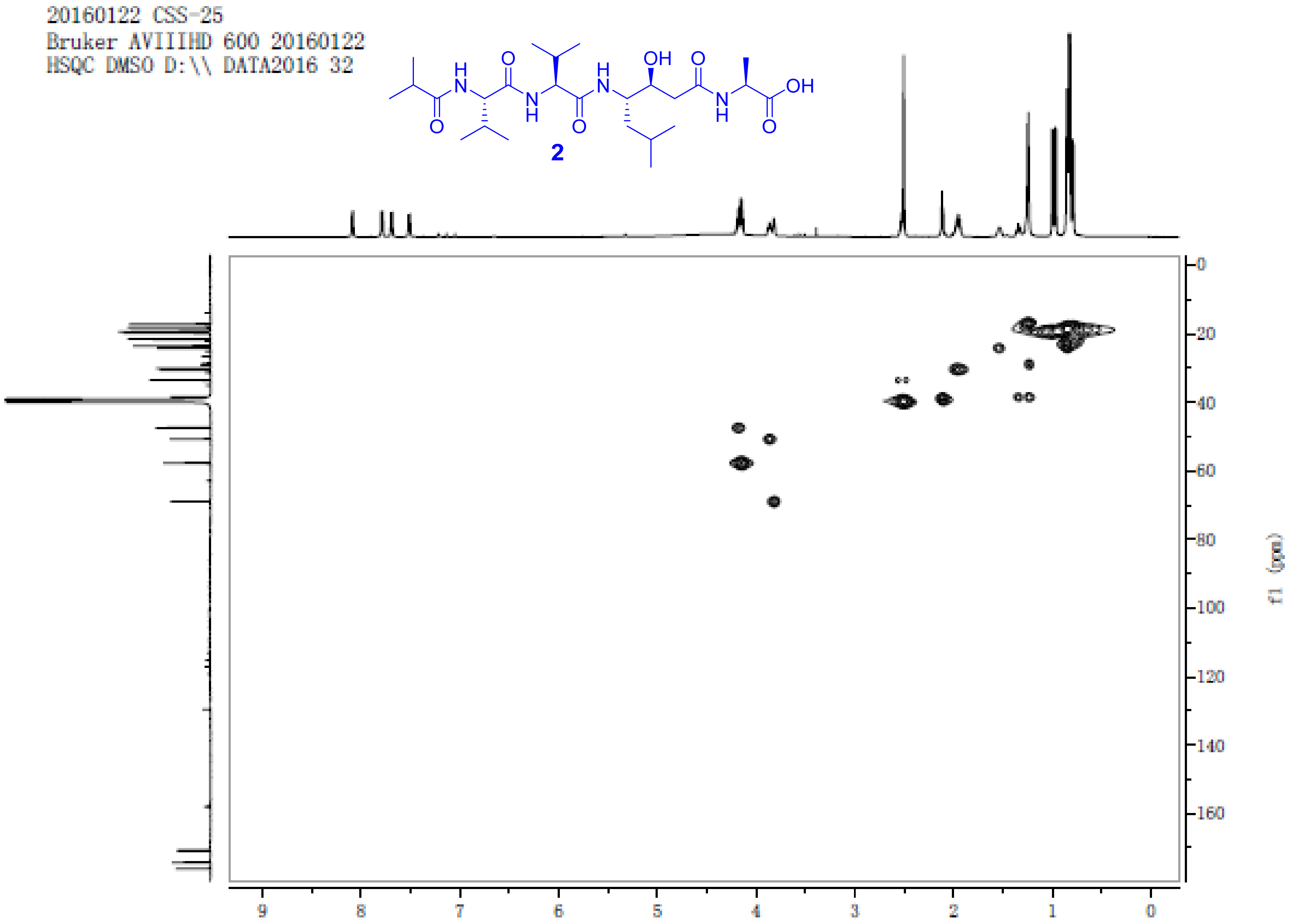

Figure S17. The HSQC Spectrum of Compound **2** in DMSO–d_6

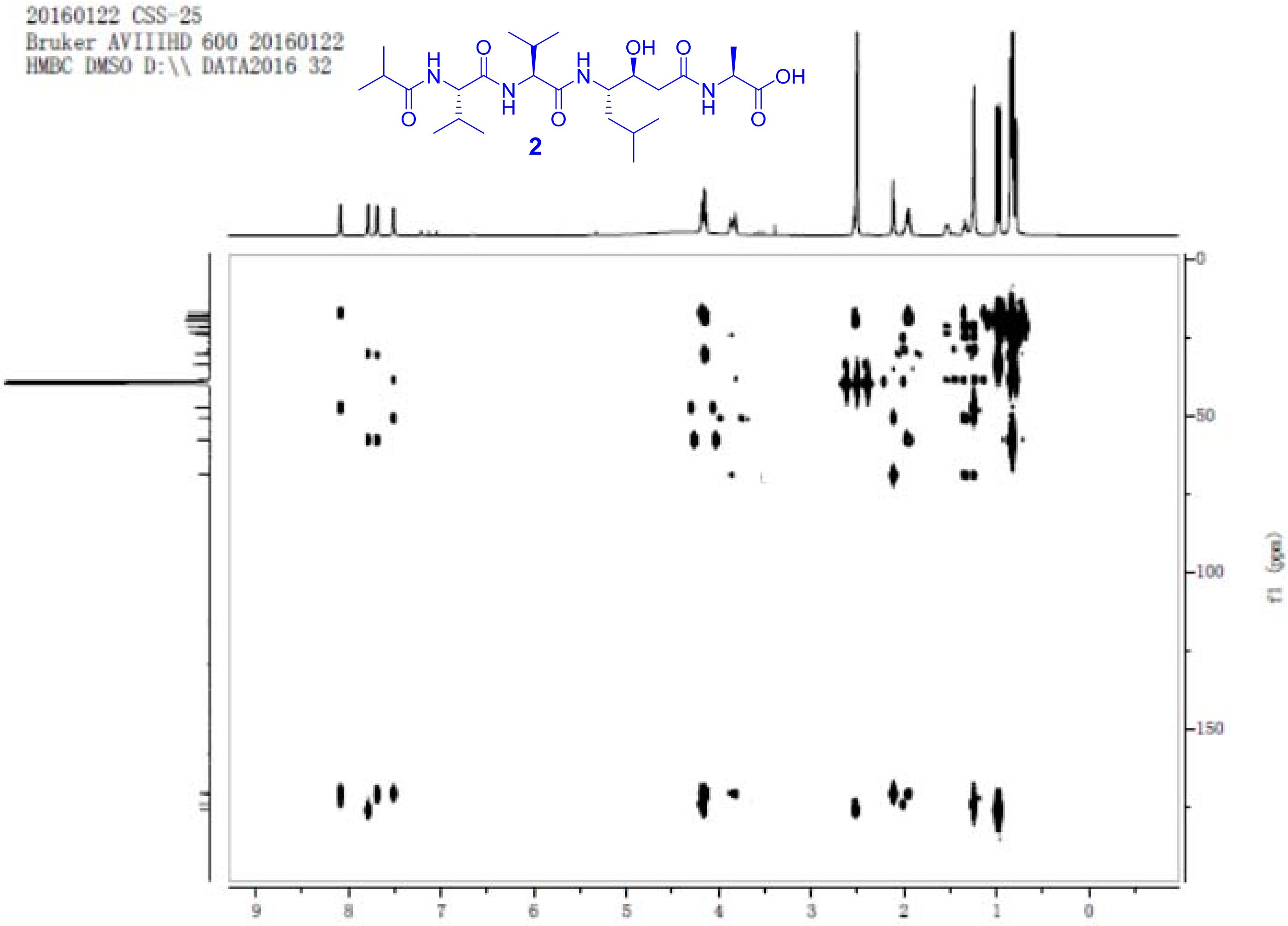

Figure S17. The HMBC Spectrum of Compound **2** in DMSO–d_6

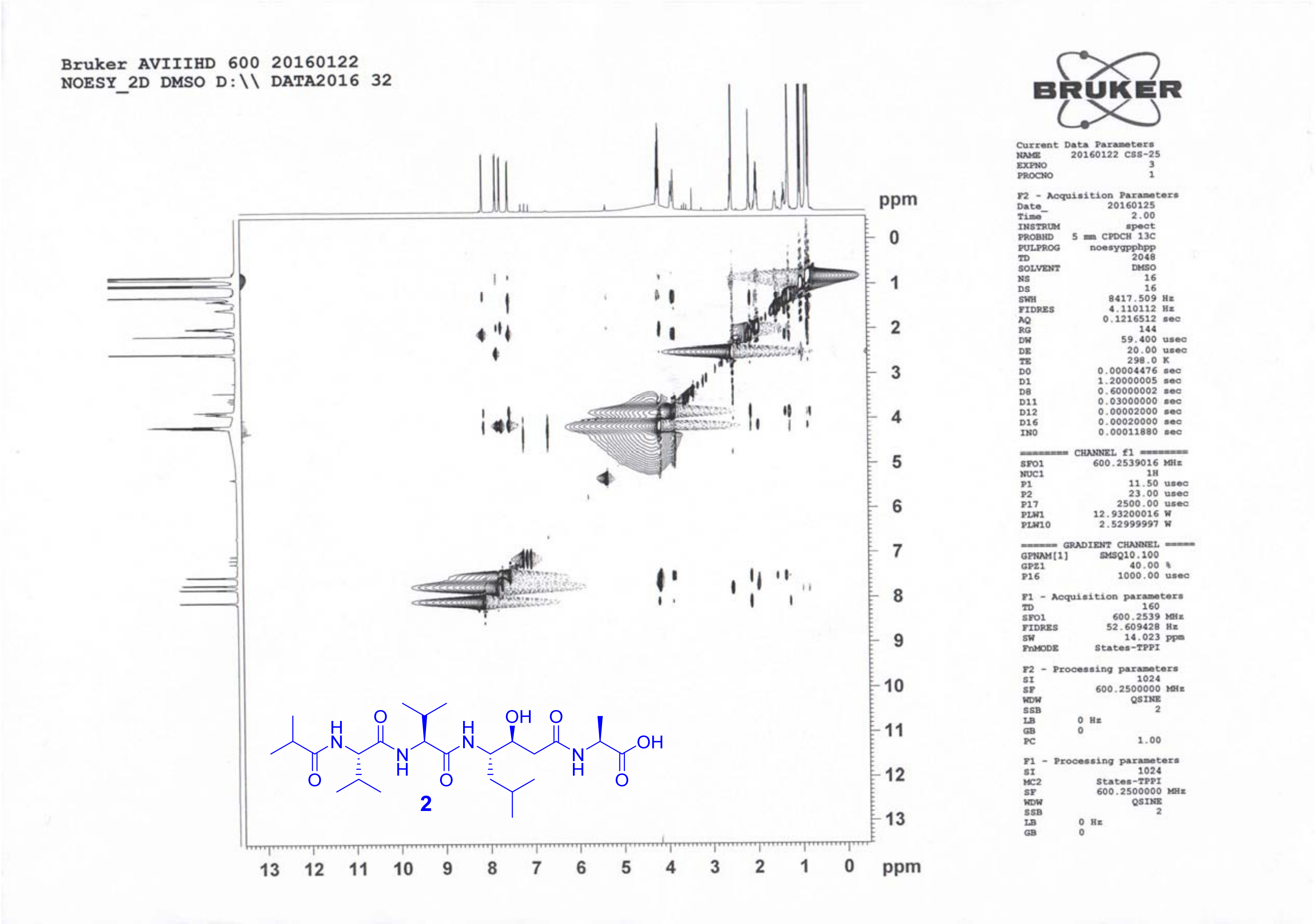

Figure S18. The NOESY Spectrum of Compound **1** in DMSO−d_6

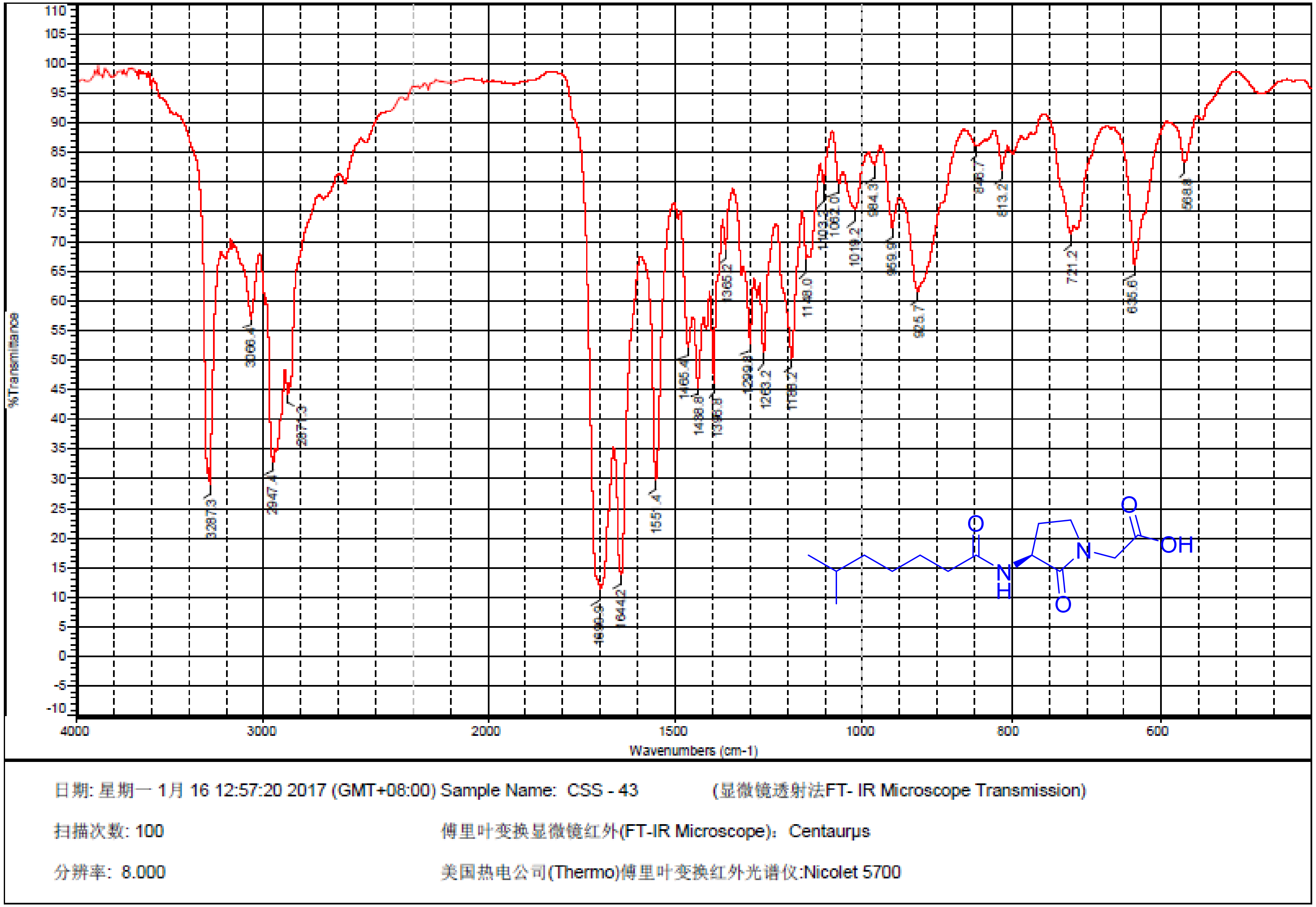

Figure S19. The IR Spectrum of Compound **3**

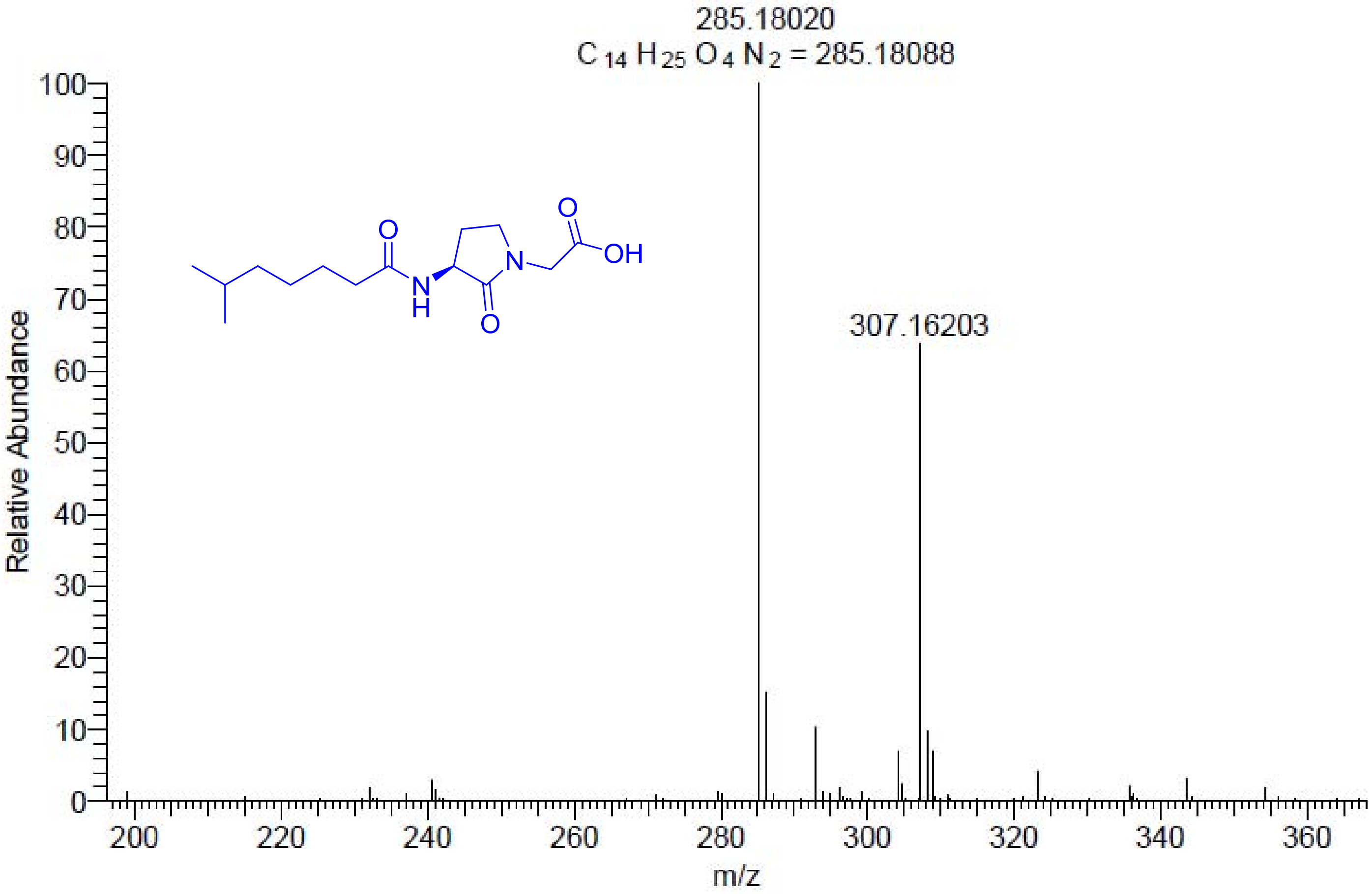

Figure S20. The (+)-HRESIMS Spectroscopic Data of Compound **3**

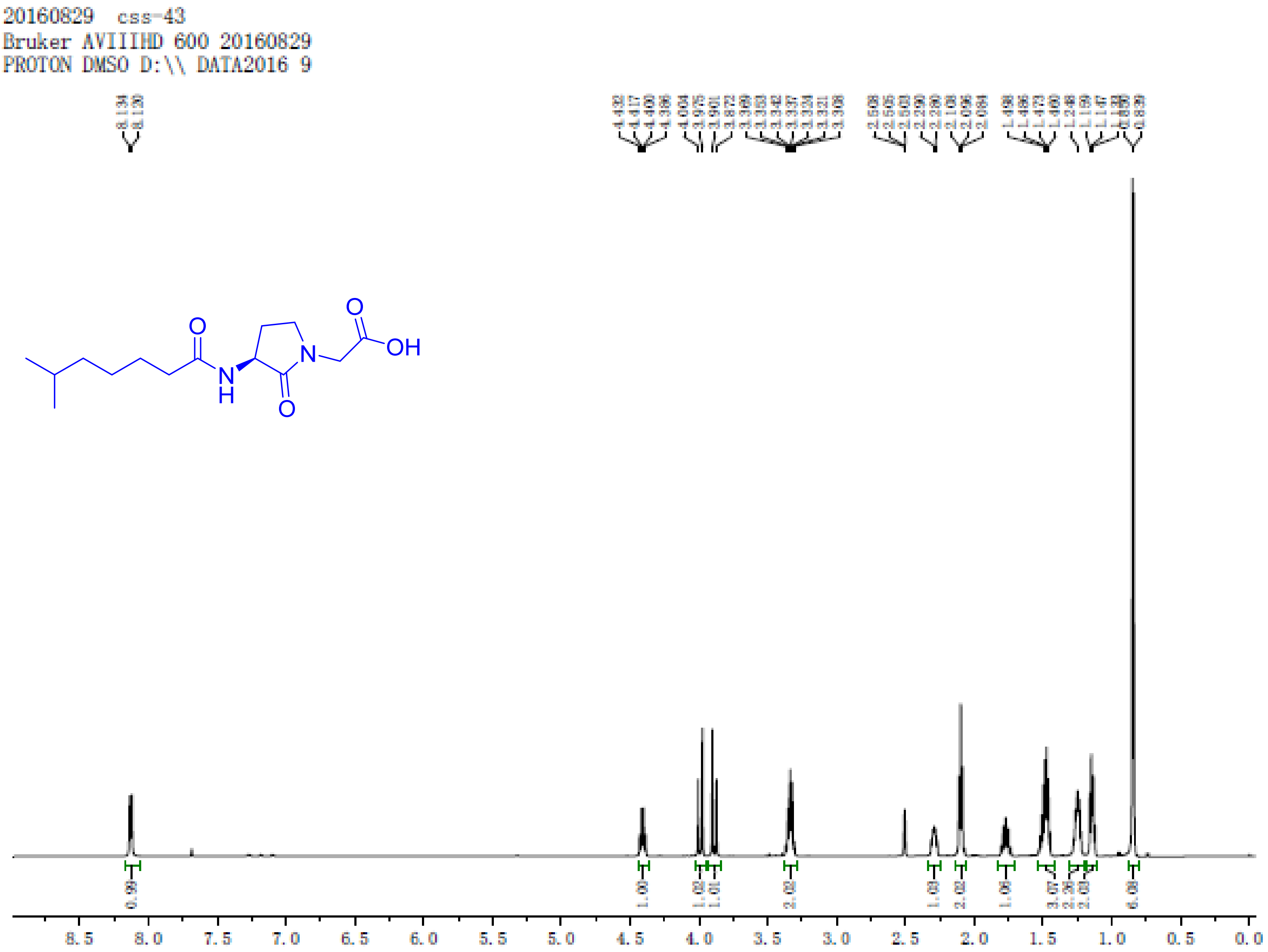

Figure S21. The ^{1}H NMR Spectrum of Compound **3** in DMSO–d_6

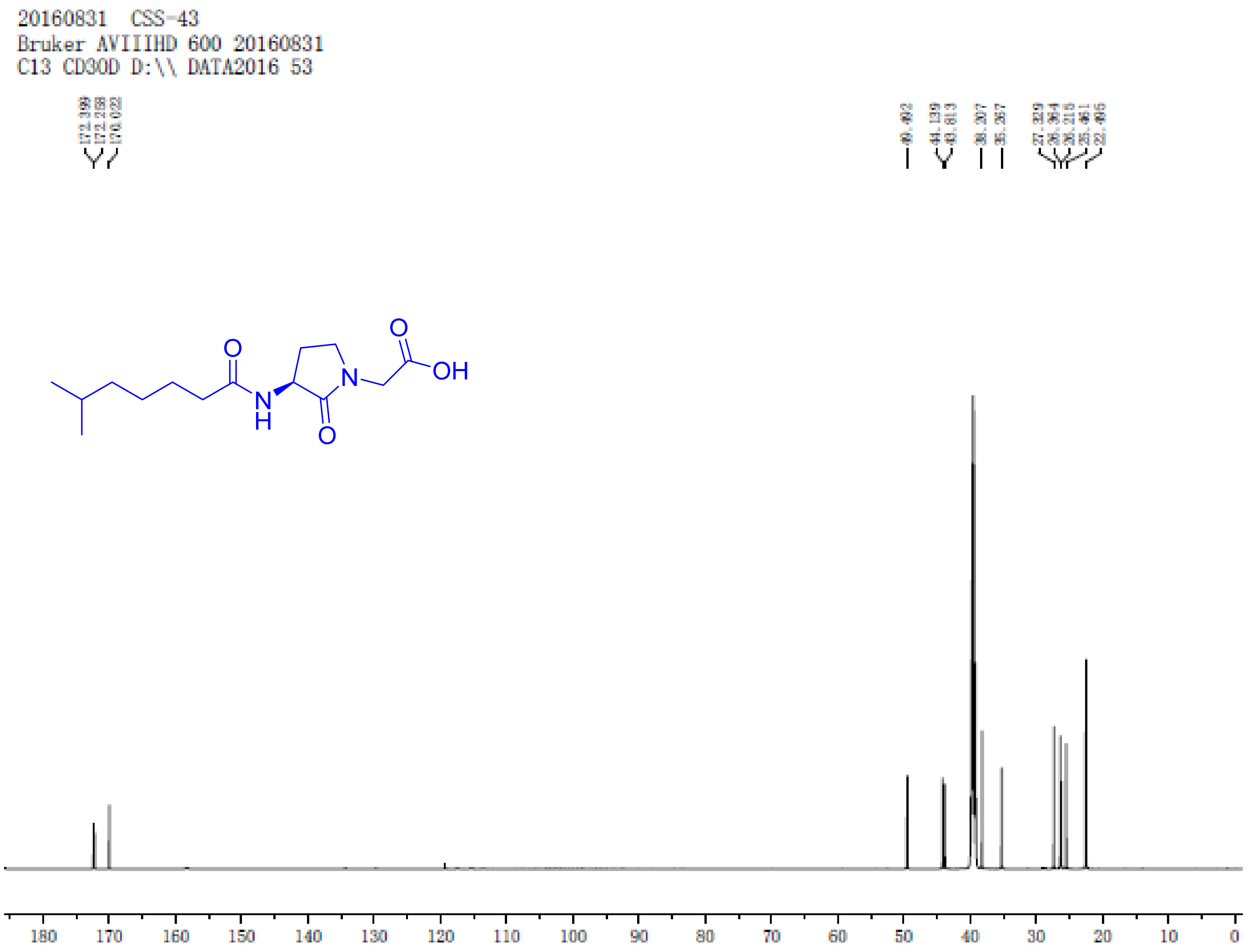

Figure S22. The ^{13}C NMR Spectrum of Compound **3** in DMSO–d_6

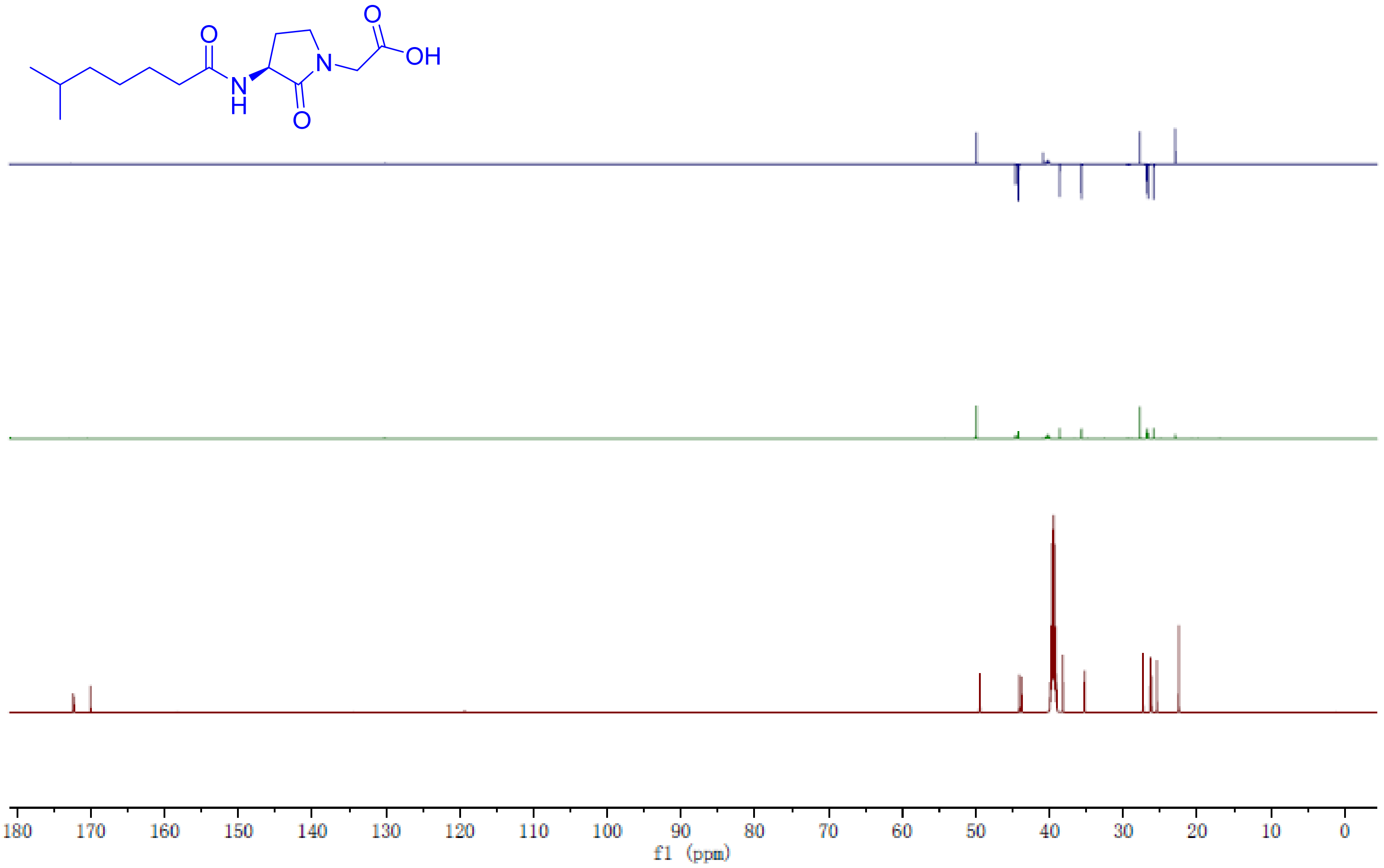

Figure S23. The DEPT Spectrum of Compound **3** in DMSO–d_6

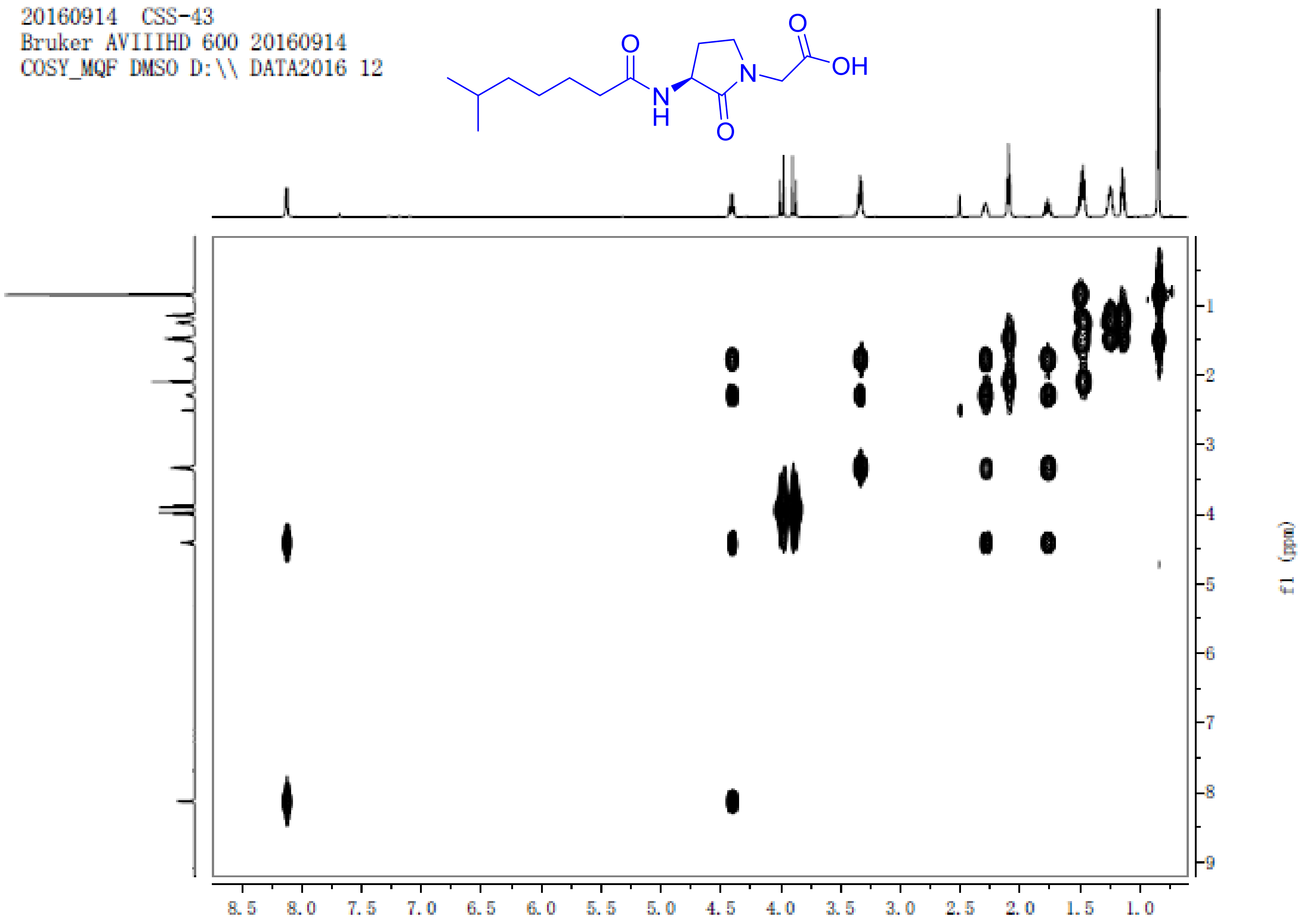

Figure S24. The ^{1}H-^{1}H COSY Spectrum of Compound **3** in DMSO–d_6

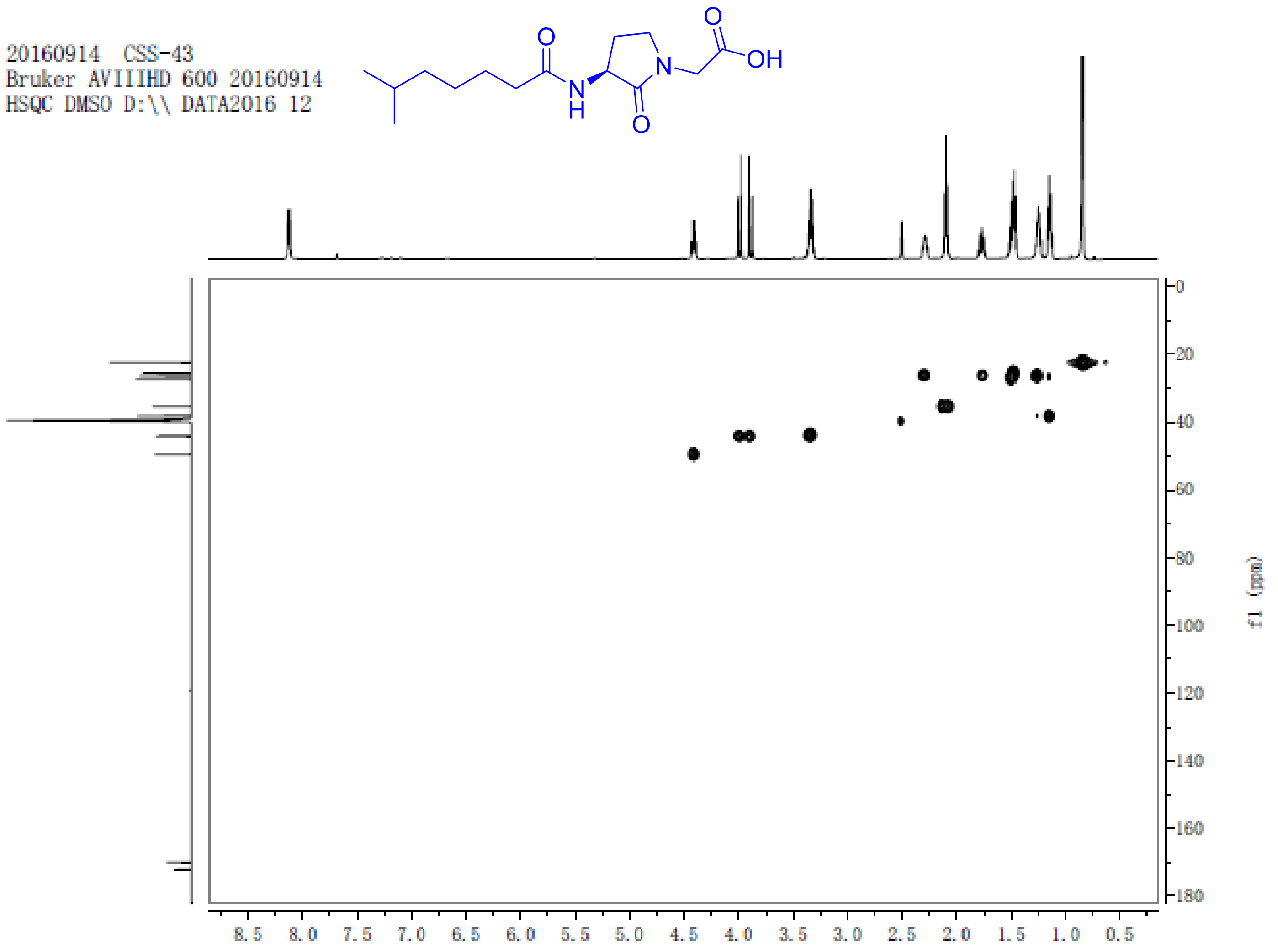

Figure S25. The HSQC Spectrum of Compound **3** in DMSO–*d*6

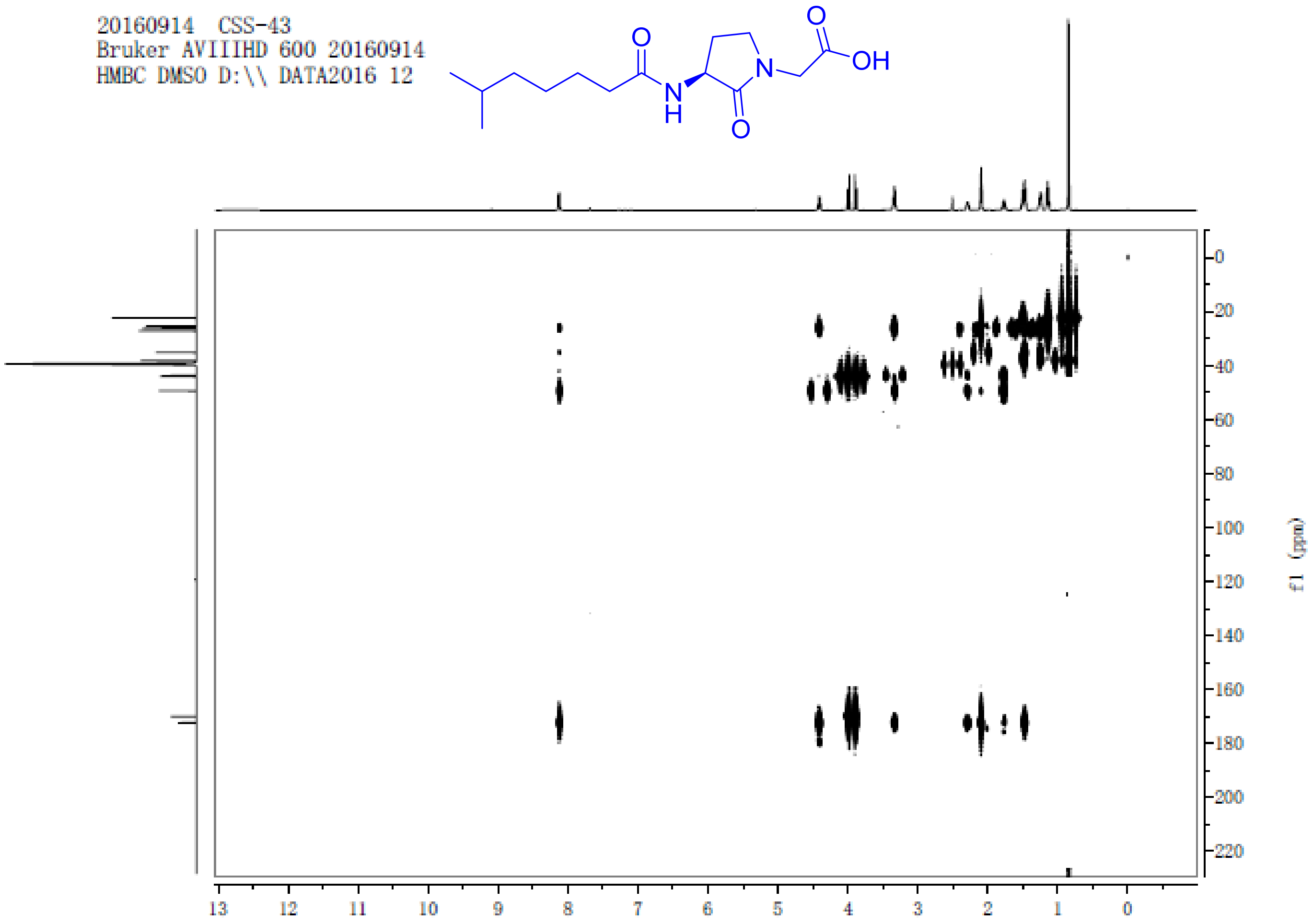

Figure S26. The HMBC Spectrum of Compound **3** in DMSO–d_6

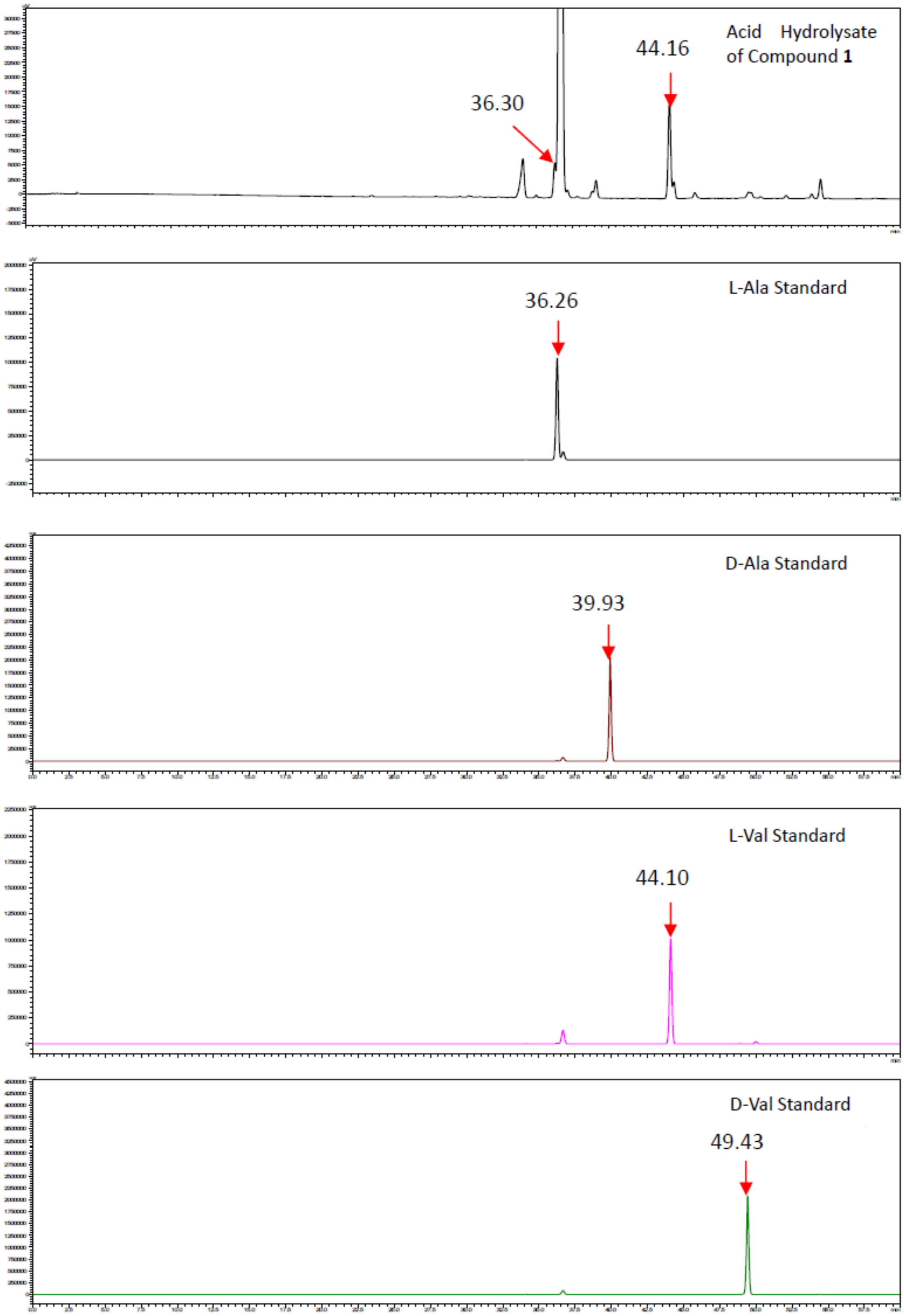

Figure S27. Marfey's Analysis of Acid Hydrolysate of Compound **1**(t_R min)

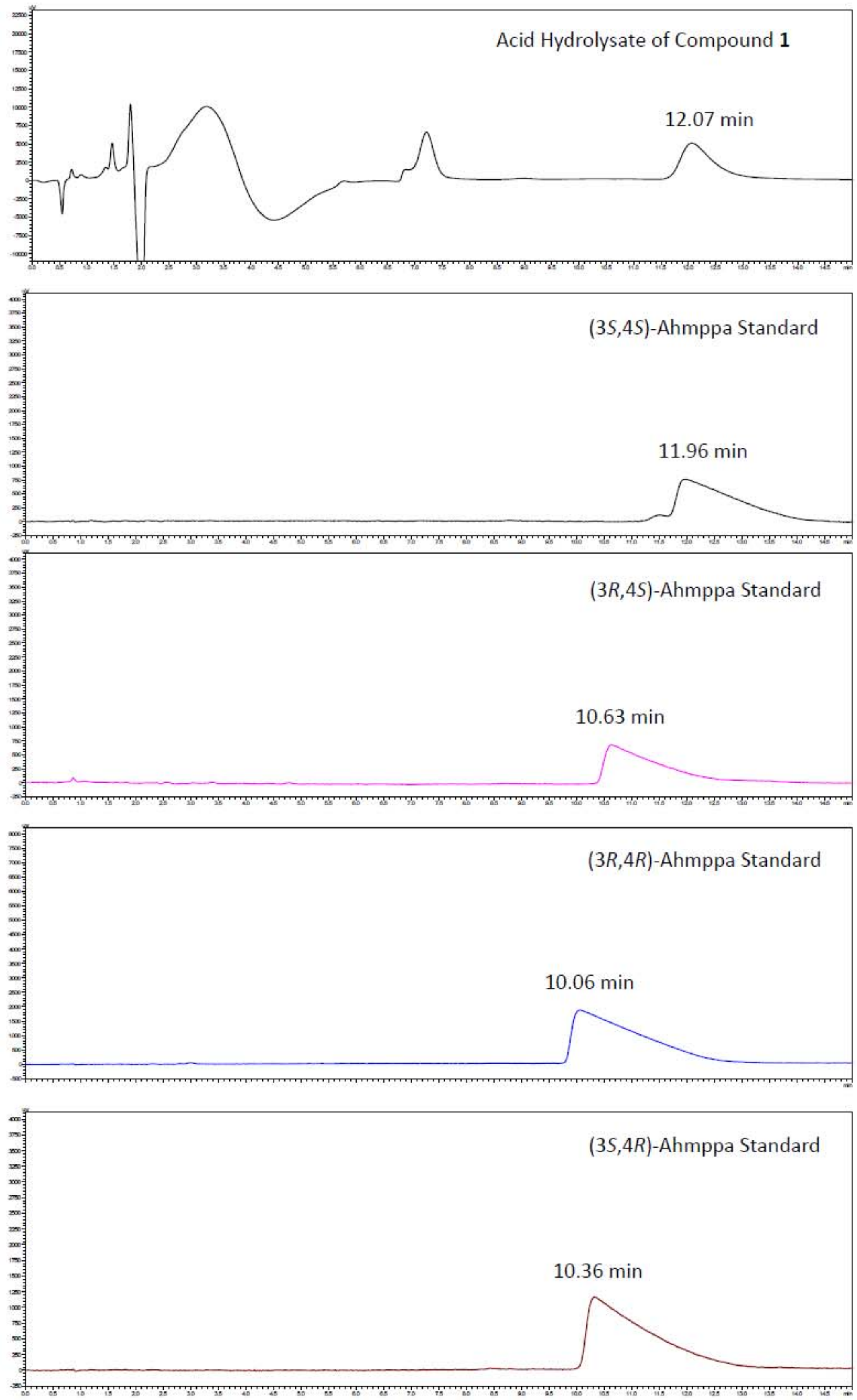

Figure S28. Chiral-phase HPLC Analysis of Acid Hydrolysate of Compound **1** (t_R min)
